# Predictable genome‐wide sorting of standing genetic variation during parallel adaptation to basic versus acidic environments in stickleback fish

**DOI:** 10.1002/evl3.99

**Published:** 2019-01-23

**Authors:** Quiterie Haenel, Marius Roesti, Dario Moser, Andrew D. C. MacColl, Daniel Berner

**Affiliations:** ^1^ Department of Environmental Sciences, Zoology University of Basel 4051 Basel Switzerland; ^2^ Biodiversity Research Centre and Zoology Department University of British Columbia Vancouver British Columbia V6T 1Z4 Canada; ^3^ Current address: Institute of Ecology and Evolution University of Bern 3012 Bern Switzerland; ^4^ Current address: Jagd‐ und Fischereiverwaltung Thurgau 8510 Frauenfeld Switzerland; ^5^ School of Life Sciences University of Nottingham Nottingham NG7 2RD United Kingdom

**Keywords:** Abiotic selection, convergence, ecological genomics, *Gasterosteus aculeatus*, North Uist, parallel evolution, Selective sweep, standing genetic variation

## Abstract

Genomic studies of parallel (or convergent) evolution often compare multiple populations diverged into two ecologically different habitats to search for loci repeatedly involved in adaptation. Because the shared ancestor of these populations is generally unavailable, the source of the alleles at adaptation loci, and the direction in which their frequencies were shifted during evolution, remain elusive. To shed light on these issues, we here use multiple populations of threespine stickleback fish adapted to two different types of derived freshwater habitats—basic and acidic lakes on the island of North Uist, Outer Hebrides, Scotland—and the present‐day proxy of their marine ancestor. In a first step, we combine genome‐wide pooled sequencing and targeted individual‐level sequencing to demonstrate that ecological and phenotypic parallelism in basic‐acidic divergence is reflected by genomic parallelism in dozens of genome regions. Exploiting data from the ancestor, we next show that the acidic populations, residing in ecologically more extreme derived habitats, have adapted by accumulating alleles rare in the ancestor, whereas the basic populations have retained alleles common in the ancestor. Genomic responses to selection are thus predictable from the ecological difference of each derived habitat type from the ancestral one. This asymmetric sorting of standing genetic variation at loci important to basic‐acidic divergence has further resulted in more numerous selective sweeps in the acidic populations. Finally, our data suggest that the maintenance in marine fish of standing variation important to adaptive basic‐acidic differentiation does not require extensive hybridization between the marine and freshwater populations. Overall, our study reveals striking genome‐wide determinism in both the loci involved in parallel divergence, and in the direction in which alleles at these loci have been selected.

**Impact Summary**

The repeated emergence of similar life forms within ecologically similar environment provides particularly convincing evidence of determinism in evolutionary diversification driven by natural selection. While well documented at the phenotypic (i.e., trait) level, the genomic underpinnings of such parallel evolution remain elusive—to what extent is phenotypic parallelism reflected by genomic parallelism, and where do the genetic variants used for repeated adaptation originate? To examine these questions, we study young (postglacial) populations of stickleback fish displaying striking phenotypic similarity within multiple basic and acidic lakes on the island of North Uist, Scotland. We first type high‐density genome‐wide single‐nucleotide polymorphisms (SNPs) five basic and five acidic populations, and in individuals from two marine sites, the latter providing a meaningful present‐day surrogate of the genomic make‐up of the marine ancestor of the lake populations. Based on these SNPs, we establish that the basic and acidic lake populations have adapted independently from one another. We then identify numerous genomic regions in which the populations show strong and consistent differentiation according to habitat, indicating widespread parallel genetic responses to divergent selection. Inspecting allele frequencies and allele associations in these regions reveals sharing of the same genetic variants within each habitat type. This adaptive genetic variation is also found in the marine ancestor, although variants selected in the ecologically relatively extreme acidic lakes tend to be uncommon in the sea. Nevertheless, these variants do not appear to be eliminated from the sea, likely because they are selectively (nearly) neutral when occurring at low frequency. Overall, our work highlights that phenotypic parallelism can be mirrored by parallel evolution at the genomic level; that the genome‐wide sorting of standing genetic variation can be predicted from the ecological difference between novel and ancestral habitats; and that considering the ancestor can greatly strengthen genomic investigations of parallel evolution.

The quest for elucidating the genomic basis of adaptive diversification commonly proceeds by comparing populations from two ecologically distinct habitat types at genome‐wide markers. Genetic loci important to differential adaptation are then identified by screening the populations for exceptionally strong habitat‐related genetic differentiation relative to the genome‐wide background level (e.g., Roesti et al. 2015; Lamichhaney et al. 2016; Reid et al. 2016; Yeaman et al. 2016; Marques et al. 2017). This approach is particularly informative when *multiple* populations adapted independently to each habitat type are available, as such “parallel” (or convergent; Arendt and Reznick 2008) evolution helps distinguish deterministic selective from stochastic genetic differentiation (Berner and Salzburger 2015).

Even deeper insights into the mechanisms of adaptation at the genomic level could, in principle, be gained by complementing the study of populations adapted in parallel to ecologically distinct habitats with genomic data from their shared ancestral population. The reason is that if information on the level of the ecological difference of each novel, derived habitat from the ancestral habitat is available, this allows generating a priori hypotheses about the direction and magnitude of selective genetic shifts away from the ancestor within each derived habitat—thus moving genomic analysis from description into the realm of prediction. Including the ancestor in genomic studies of multiple derived populations further offers the advantage that the origin of the polymorphisms under divergent selection between the derived habitats can be explored directly. An obvious obstacle to such genomic investigation, however, is that natural systems providing access to the ancestor of populations adapted in parallel to multiple derived habitats are rare.

We here adopt this uncommon analytical perspective in a genomic investigation of threespine stickleback fish from North Uist, an island of the Outer Hebrides, Scotland. Starting from a common marine ancestral population, stickleback have independently colonized numerous lakes on North Uist 8000–10,000 years ago (Fig. 1A; Campbell and Williamson 1979; Ballantyne 2010). Because of a sharp transition in surface geology, lakes in the west of the island display a basic pH and are meso‐ to eutrophic, whereas lakes in the east are consistently acidic, oligotrophic, and relatively depleted in dissolved ions (e.g., the calcium concentration is 10 times higher in the basic lakes than in the acidic lakes on average; Supporting Information Table S1). These ecological differences have proved stable over decades of investigation (Waterston et al. 1979; Giles 1983; Spence et al. 2013; Klepaker et al. 2016; Magalhaes et al. 2016). The difference in water chemistry between these two lake types, hereafter simply referred to as “basic” and “acidic,” mirrors distinct levels of ecological difference from the ancestral marine habitat, with acidic lakes being more different from the sea than the basic lakes (visualized for pH and calcium concentration in Fig. 1B, top panels). Accordingly, basic and acidic lake populations have evolved different levels of phenotypic differentiation from their marine ancestor. For instance, marine stickleback generally exhibit long pelvic and dorsal spines and extensive lateral plating along most of their body, bony armor serving as protection from predators (Bell and Foster 1994). By contrast, typical freshwater ecotypes, including those in the basic lakes of North Uist, have their armor reduced to a bony girdle consisting of the pelvic complex and dorsal spines interconnected by only a few lateral plates (Fig. 1B and C; detailed data provided in Supporting Information Table S1). In stickleback ecotypes from the acidic lakes, this armor reduction has progressed further to an extreme level. Here, the pelvic complex, dorsal spines, and lateral plates are either rudimentary or missing altogether. Striking parallel evolution has also occurred in body size and shape (Fig. 1C; Campbell and Williamson 1979; Giles 1983; MacColl et al. 2013), with the dwarf stickleback residing in the acidic lakes ranking among the smallest vertebrates in Europe.

**Figure 1:**
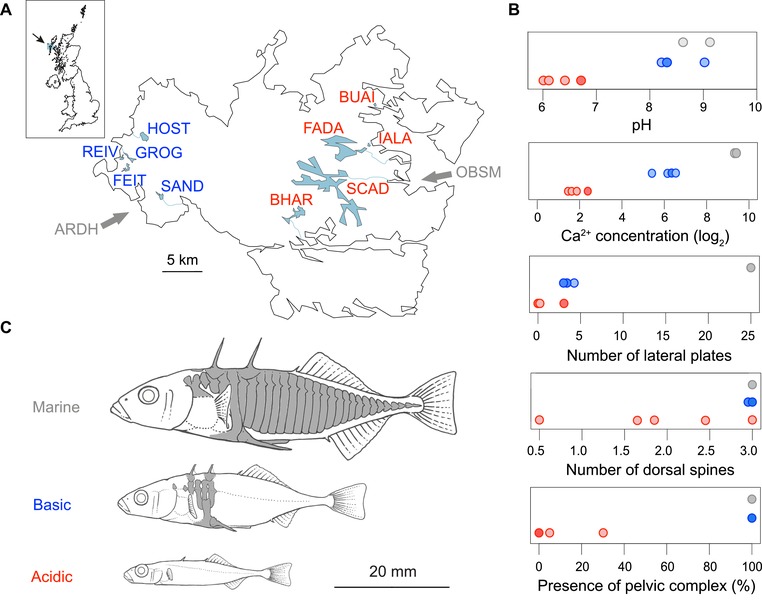
Stickleback study populations and their habitats. (A) Geographic situation of the basic (blue) and acidic (red) lakes on North Uist, Outer Hebrides, Scotland, with their connections to the sea shown as fine blue lines (the outlet of FEIT is uncertain). The gray arrows indicate the two coastal lagoons where marine stickleback were sampled. The same habitat‐specific color coding is used throughout the paper. (B) pH, calcium (Ca^2+^) concentration (in mg/L), and armor trait (lateral plate and dorsal spine counts, presence of pelvic spines and pelvic complex) mean values across individuals for each study site (data presented in detail in Supporting Information Table S1). The data points are arranged vertically according to habitat type, and they sometimes overlap (especially in the phenotypically uniform marine fish; overlap is indicated by darker dots). Data on pH and calcium concentration are from Magalhaes et al. (2016). Calcium measurements were log_2_ transformed. (C) Typical stickleback ecotypes from the three focal habitats, drawn to relative scale. Elements of bony armor are shaded in gray, including the dorsal spines, lateral plates, and the ventral pelvic complex to which the pelvic spines attach.

Based on the ecological differences between the sea, the basic lakes and the acidic lakes, and the concurrent phenotypic parallelism exhibited among the derived populations within each freshwater habitat, we ask two main questions guiding our genomic investigation: first, is parallelism in the evolution of basic and acidic lake stickleback ecotypes mirrored in the sharing of distinctive adaptive genetic variants within each lake habitat type? Despite its simplicity and importance to understanding the genomic basis of evolution, the congruence in parallelism at the phenotypic and genotypic levels remains poorly evaluated empirically in higher organisms. The reason is that genomic investigations of natural systems exhibiting phenotypic parallelism commonly lack the marker resolution needed to achieve robust conclusions about parallelism at the genomic level (Berner and Roesti 2017; Lowry et al. 2017; Haenel et al. 2018; for exceptions, see Martin et al. 2013; Lamichhaney et al. 2016; Reid et al. 2016; Yeaman et al. 2016; Elgvin et al. 2017). Second, has the greater ecological difference of the acidic than basic lakes from the ancestral habitat caused asymmetry in the selection of adaptive genetic variation? We expect that stickleback in acidic lakes should have adapted to their extreme habitat by accumulating alleles relatively rare in their ancestor, while at the same loci, the populations in basic lakes should have retained alleles also occurring at high frequency in the sea. This idea is amenable to empirical examination because present‐day marine stickleback living around North Uist provide a proxy for the ancestor of all derived freshwater populations on the island. Using high‐resolution single‐nucleotide polymorphism (SNP) data from samples from all three habitats, we confirm both genomic parallelism and habitat‐related asymmetry in the selection of standing genetic variation, thus uncovering a strongly deterministic component to adaptive diversification at the genomic level.

## Methods

### STICKLEBACK SAMPLES

Freshwater stickleback were captured from five basic and five acidic lakes on North Uist (Fig. 1A) during the 2014 breeding season (mid‐April to late May), aiming for a sample size of 30 individuals per lake (details given in Supporting Information Table S1). The lakes were chosen to represent separate watersheds draining independently into the sea, although for one basic lake, this could not be determined unambiguously (FEIT; this lake may reside in the same watershed as GROG, although a direct present‐day connection can be ruled out). Marine stickleback were sampled on breeding grounds in two tidal lagoons located on the east coast (OBSM, *N* = 20, sampled 2013) and west coast (ARDH, *N* = 10, sampled 2016) of the island. These fish, however, were not lagoon‐residents—which exist on North Uist but are phenotypically distinct (El Nagar and MacColl 2016)—but truly anadromous marine stickleback. Marine stickleback exhibit large population sizes and are genetically well mixed over large distances (Hohenlohe et al. 2010; Jones et al. 2012a; Catchen et al. 2013; Roesti et al. 2014; Lescak et al. 2015), and they inhabit a relatively constant habitat, so that present‐day marine samples are generally considered meaningful surrogates of the ancestor of nearby derived freshwater populations. All sampling was performed with unbaited minnow traps. Specimens were euthanized with an overdose of MS222 and immediately preserved in absolute ethanol. Details on the sampling locations and habitats are given in Supporting Information Table S1 (see also Giles 1983; MacColl et al. 2013; Spence et al. 2013; Klepaker et al. 2016; Magalhaes et al. 2016).

### PHENOTYPIC ANALYSES

To highlight parallelism in phenotypic evolution among our study populations from each habitat type, we scored armor traits known to exhibit strong variation among North Uist freshwater stickleback, presumably driven by selection associated with predation and differences in water chemistry (Giles 1983; MacColl et al. 2013; Spence et al. 2013; Klepaker et al. 2016; Magalhaes et al. 2016). These traits were chosen for ease of measurement, recognizing that selection related to water chemistry has likely targeted numerous life history and physiological traits beyond external bone morphology. Twenty total individuals chosen at random from each lake sample, and all individuals from the two (smaller) marine samples, were scored under a dissecting microscope for the number of lateral plates (right body side), number of dorsal spines, presence (at least as rudiment) or absence of the pelvic complex and of the pelvic spines. Counts were averaged for each population.

### DNA LIBRARY PREPARATION AND SEQUENCING

To obtain genetic markers, we first extracted DNA individually from fin tissue of each of the 288 total stickleback from the 10 total freshwater populations (Supporting Information Table S1) by using a MagNA Pure LC278 extraction robot (Roche, Basel, Switzerland) and the Tissue Isolation Kit II. After an RNase treatment, DNA concentrations were standardized to 10 ng/μL based on two rounds of Qubit (Invitrogen, Thermo Fisher Scientific, Wilmington, DE, USA) quantitation, and used to prepare high‐resolution pooled restriction site‐associated DNA (RAD). Specifically, 3.3 μL of adjusted DNA solution from each individual of a given population were transferred to each of two replicate sample pools. Each of these two pools per population was then split further into two subpools of 50 μL subjected to restriction with either the *Nsi1* enzyme (approximately 164,000 recognition sites across the 460 megabases [Mb] stickleback genome) or the *Pst1* enzyme (314,000 recognition sites) (New England Biolabs, Ipswich, MA, USA). The rationale of the parallel restriction of each subpool with a separate enzyme was to avoid DNA fragments too short for sequencing that would have resulted from the *simultaneous* restriction with *Nsi1* and *Pst1* at recognition sites located in close proximity. Our parallel‐restriction approach (see Supporting Information Fig. S1 for a schematic) thus allowed interrogating the stickleback genome at approximately 478,000 total restriction sites, resulting in a 22 times higher physical resolution than what would be achieved by using the standard *Sbf1* enzyme. The two digested subpools of each pool were then labeled with the same molecular barcode (four barcodes used in total; two 5mer, one 6mer, and one 7mer) and then combined, yielding two replicate pools per population. These pools were then subjected to the standard RAD library preparation steps (Baird et al. 2008). Enrichment polymerase chain reaction (PCR) occurred in seven replicate reactions per library (i.e., pool) to reduce amplification bias. The 20 total libraries were single‐end sequenced to 200 base pairs on five lanes of an Illumina HiSeq2000 instrument, always allocating the two replicate libraries of a given population to different lanes.

DNA from the 30 total individuals from the two marine samples was extracted (Qiagen DNeasy Blood & Tissue Kit Valencia, CA, USA) and barcoded individually, pooled PCR‐free into a single library, and whole‐genome (not RAD) paired‐end sequenced to 151 base pairs on a single Illumina HiSeq2500 lane.

### MARKER GENERATION

Raw sequence reads were parsed by barcode (i.e., population), pooled over the two replicate libraries, and aligned to the third‐generation assembly (Glazer et al. 2015) of the stickleback reference genome (Jones et al. 2012b) by using Novoalign (Version 3.0, http://www.novocraft.com/products/novoalign/; alignment settings provided on the Dryad repository, https://doi.org/10.5061/dryad.4ck2q0m). Resulting SAM files were converted to BAM format and accessed in R using Rsamtools (Morgan et al. 2017). SNPs were ascertained in the global freshwater pool (i.e., all basic and acidic populations combined), requiring a total read coverage between 150 and 2800 (the latter effectively filtering sequences from repeated elements), a minor allele frequency (MAF) superior to 0.05 across the pool, and a distance to the nearest polymorphism of at least 12 base pairs (effectively avoiding microindel stutter). A total of 253,451 SNPs passed these filters, yielding an approximate average resolution of 1 SNP per 2 kilobases (kb)—higher than in any previous reduced‐representation sequencing study (Lowry et al. 2017). At these SNPs, we performed nucleotide counts for each freshwater population at an average read depth of 63× per population pool. At the same SNPs, we then also performed nucleotide counts for the two marine samples (for which full‐genome data were generated; Supporting Information Fig. S1). For analysis, nucleotide counts from all samples were stored in a single SNP matrix (available on Dryad). To achieve the standard individual‐level sample size of the lake populations, SNP data from the two genetically very similar (Supporting Information Tables S2 and S3) marine samples were combined to a single population (average read depth: 133×) in all analyses except the phylogenies and ordination (a detailed justification for combining the two marine samples to a single population is provided in the Supporting Information “Discussion” section, paragraph 1).

### GENETIC SIMILARITY AMONG POPULATIONS

A fundamental requirement in investigations of parallel evolution is evidence that the focal populations have adapted to their habitat independently from other, ecologically similar populations (Endler 1986; Schluter 2000). Our study lakes presently reside in separate watersheds draining independently into the sea (Fig. 1A). Moreover, a previous genetic investigation including a subset of our study populations suggests their evolutionary independence (Magalhaes et al. 2016). To extend this evidence to all our study populations, we first characterized their genetic similarity by nuclear phylogenies. For this, we filtered the SNP matrix for SNPs occurring alone on a RAD locus (i.e., “loner” SNPs sensu Roesti et al. 2015; to maximize their independence) and exhibiting a base coverage of at least 40× within each population pool. To minimize the influence of selection, we further excluded SNPs showing substantial allele frequency differentiation (>0.5) in both the combined basic‐acidic and marine‐freshwater differentiation scans (details below). Moreover, a SNP had to reside within 5 Mb from the nearest tip of the corresponding chromosome—a genomic region exhibiting a particularly high recombination rate (Roesti et al. 2013; Glazer et al. 2015). Since we sequenced pooled DNA and hence individual genotypes were not available, we used the resulting 15,058 SNPs to generate 10 synthetic diploid genotypes for each population by drawing nucleotides at random without replacement from the corresponding population pool and concatenating them after translation to IUPAC ambiguity code. We next inferred the most appropriate model of sequence evolution (“GTR+G+I”) using the R package *phangorn* (Schliep 2011) and constructed a maximum likelihood tree (neighbor‐joining produced similar results in all phylogenetic analyses). In this analysis, all 10 freshwater populations proved reciprocally monophyletic—consistent with the absence of admixture inferred from individual‐level genotype data from a subset of our populations including all acidic ones (Fig. 3 and Supporting Information Fig. S1 in Magalhaes et al. 2016), so we present a simplified tree based on a single individual per population only (data provided in fasta format on Dryad; the tree based on the full samples is shown in Supporting Information Fig. S2). Additional phylogenies were performed by expanding the dataset to *all* loner SNPs satisfying the above base coverage criterion (68,245 SNPs), and by restricting the dataset to loner SNPs separated by at least 1 Mb (227 SNPs). Over this latter physical distance, linkage disequilibrium is minimal in this (e.g., Roesti et al. 2015) and many other species (Lowry et al. 2017) so that synthetic multilocus genotypes should resemble natural genotypes (further justification for using synthetic genotypes for phylogenetic inference is elaborated in the Supporting Information “Discussion” section, paragraph 2).

In a second analysis, we explored the genetic similarity among our populations by ordination using nonmetric multidimensional scaling (NMDS) and the stringently filtered dataset described above (15,058 SNPs). At each SNP, we first identified the major and minor allele across the global allele pool comprising all freshwater populations. Next, we randomly sampled a single allele from each population at each SNP, assigned these alleles the value of 1 (major allele) or 0 (minor allele), and derived a binary population similarity matrix from these values using the R function *dist*. Finally, we extracted ordination coordinates from the similarity matrix using the function *isoMDS* (good fit was achieved with seven dimensions; stress = 0.06), and visualized the populations along the first two. Running a principal component analysis on the same data and visualizing the populations along the first two components produced almost identical results.

### IDENTIFYING LOCI UNDER PARALLEL BASIC‐ACIDIC DIFFERENTIATION

A major objective of our study was to assess if the repeated evolution of characteristic ecotypes within the basic and acidic lakes is mirrored by the consistent, parallel sorting of genetic variation between these habitats. Our key resource to address this question were genome‐wide scans for the magnitude of genetic differentiation performed for all 45 possible pairwise population comparisons within and across the freshwater habitat types. These included 25 basic‐acidic (B‐A), 10 basic‐basic (B‐B), and 10 acidic‐acidic (A‐A) population combinations. We here considered only SNPs displaying a total read coverage of at least 50× within each population, and a MAF across the global pool of all B and A populations superior to 0.2 to ensure adequate information content (Roesti et al. 2012). Although not the focus of the present study, we also performed an analogous genome‐wide differentiation scan by treating all lake populations simply as freshwater stickleback, and comparing them to our marine population (i.e., a standard analysis of parallel evolution in marine‐freshwater stickleback; e.g., Hohenlohe et al. 2010; Jones et al. 2012a; Roesti et al. 2014; Terekhanova et al. 2014). As a resource, this latter analysis is presented as Supporting Information Figure S3 and the underlying SNP data (ascertained differently than our main SNP dataset) are provided on Dryad.

Population differentiation, quantified by the absolute allele frequency difference (AFD), was then integrated within each of the three freshwater habitat comparison categories. To do so, we calculated at each SNP the mean of the AFD values from all population pairs in a given comparison category, provided the SNP was represented by a sufficient number of individual comparisons (thresholds: at least 18 for B‐A, at least 8 for B‐B and A‐A). (Genome‐wide mean and median differentiation values for all pairwise population and habitat type comparisons, quantified by both AFD and F*_ST_*, are presented in Supporting Information Tables S2 and S3, and the underlying raw pairwise comparisons are available on Dryad). This averaging did not involve adjusting AFD values from a given population comparison by the corresponding overall level of differentiation, although performing such standardization did not materially affect our results (Supporting Information Fig. S4). Integrated this way, the AFD data were screened for genomic regions displaying exceptionally strong and consistent differentiation in the B‐A comparison category (note that this approach necessarily precludes conclusions about genomic regions involved *inconsistently* in adaptation within an ecotype; see Discussion S2 in Roesti et al. 2014). We identified genomic regions of extreme B‐A differentiation based on all SNPs exceeding an AFD threshold of 0.70, corresponding to the 99.95 percentile of the AFD distribution across all genome‐wide SNPs in this comparison category (204,433 SNPs). When located on the same chromosome, a high‐differentiation SNP was considered to represent an independent genome region when separated by at least 50 kb from any other such SNP. We hereafter refer to the single most strongly differentiated SNP within each high‐differentiation region thus identified as “core SNP.” In a supplementary analysis, the exactly same SNPs underlying our integrated B‐A comparison were subjected to a search for habitat‐associated outliers using BayPass (Gautier 2015), which generally identified similar genomic regions as our method (Supporting Information Fig. S5A). We next retrieved all genes located within a 100 kb window centered at each core SNP from the reference genome annotation, along with their functions as specified by the Ensembl and GeneCards data bases. This information was not subjected to a formal candidate gene analysis, but inspected qualitatively for genes appearing particularly likely to be involved in bone evolution, or having emerged as candidate adaptation genes in previous stickleback work.

To support the reliability of our search for genomic regions involved in parallel B‐A differentiation based on pooled RAD sequencing and the averaging of multiple population comparisons, we performed targeted individual‐level Sanger sequencing at two top core SNPs identified by the above genome scans. For both regions, we amplified a 700 bp fragment from a subsample of 4–8 individuals per sample site (Supporting Information Table S1), using primers and PCR conditions described in the Supporting Information “Methods”. The resulting sequences were aligned and screened for SNPs using Geneious version 11.1.2, and haplotype reconstruction was performed using PHASE version 2.1 (Stephens et al. 2001). Genealogies were then constructed with RAxML version 8 (Stamatakis 2014) and visualized as haplotype networks in FITCHI (Matschiner 2016) by collapsing haplotypes separated by less than three edges (–e 3 option).

### CHARACTERIZING THE ALLELES UNDER PARALLEL BASIC‐ACIDIC DIFFERENTIATION

Acidic lakes show a greater ecological difference from the sea than basic lakes, and acidic ecotypes display stronger phenotypic differentiation from their marine ancestor than basic ecotypes (Fig. 1B and C). Our second main expectation was thus that at loci showing parallel B‐A differentiation, the acidic ecotypes should generally have recruited alleles relatively unfavorable and hence rare in the marine ancestor, whereas the basic ecotypes should have retained alleles common in the ancestor. This prediction was investigated by three approaches based on the core SNPs identified as described above (*N* = 42). The first approach was phylogenetic and involved deriving a single diploid multilocus genotype for each population by sampling two nucleotides at random from each population‐specific pool at each core SNP, and concatenating them as IUPAC characters. The resulting data were used to construct a maximum likelihood tree as described above. We then repeated this procedure for the same number of SNPs (*N* = 42) chosen at random from the genome‐wide SNP panel. To ensure that these latter “random SNPs” were minimally affected by divergent selection between the basic and acidic lakes, we here only considered SNPs exhibiting a magnitude of differentiation within 0.5% of the median value (AFD = 0.25) observed across all SNPs in the combined B‐A comparison. Our prediction in this phylogenetic analysis was that at the core SNPs, the basic populations should show a greater genetic similarity to the marine fish than the acidic populations, whereas in the genealogy for the random SNPs, no freshwater ecotype should appear systematically closer to the marine fish.

Our second approach to investigating if the acidic ecotypes are genetically more derived from their marine ancestor than the basic ones involved ordination using NMDS. We here followed the protocol described above, except that only a single ordination axis was extracted from both the core and random SNPs. Our prediction was that at the core SNPs only, the basic populations should display a greater genetic similarity, and hence greater proximity on the NMDS ordinate, to the marine fish than the acidic populations.

The third approach, finally, was a locus‐specific analysis of allele frequencies at the core SNPs. We here first classified the two alleles at each SNP as “basic” or “acidic,” based on their average frequency over all populations within each lake type. That is, the allele exhibiting an average frequency >0.5 across the basic populations was considered the basic allele, and vice versa. Then we determined the frequency of the basic and acidic allele at each SNP in the marine population, which allowed us to evaluate the prediction that core SNP alleles characteristic of the acidic ecotypes occur at relatively low frequency in the marine ancestor. We here again used the random SNPs as a negative control, determining basic and acidic alleles as described for the core SNPs.

As a robustness check, all analyses described in this section were repeated with an independent sample of random SNPs selected by controlling their magnitude of differentiation in the B‐A comparison less strictly. This produced very similar results supporting the same conclusions (Supporting Information Fig. S6).

### ANALYSIS OF SELECTIVE SWEEPS

Observing that core SNP alleles typical of acidic ecotypes tended to be less common than basic alleles in the ancestral habitat (see “Results and Discussion” section), we finally hypothesized that genetic diversity should be relatively reduced around the core SNPs in the acidic populations. The reason is that in these populations, the locally favorable variants must generally have experienced greater frequency changes reducing neutral variation in the physically linked chromosomal neighborhood particularly effectively (i.e., stronger selective sweeps) (Maynard Smith and Haigh et al.^1974^). To explore this idea, we quantified genetic diversity within each lake population as the total number of SNPs with a MAF >0.3 across the 40 kb window surrounding a given core SNP. A high MAF threshold was chosen because selective sweeps shift the MAF distribution downward (Braverman et al. 1995), hence the density of high‐MAF SNPs should be particularly sensitive to sweeps. A supplementary analysis comparing the density of high‐MAF SNPs to nucleotide diversity (π) as measures of genetic diversity confirmed this expectation, and further revealed that the former is highly robust to prefiltering SNP data with mild MAF thresholds whereas nucleotide diversity can become strongly biased by such filtering (Supporting Information Fig. S7). The SNP count obtained was then summed over all populations within each lake category and divided by the analogous sum of SNPs observed across a larger (1 Mb) window around the same core SNP. The latter standardization served to adjust for general differences in genetic diversity between the ecotypes. For the relative “SNP density” metric thus obtained for each core SNP, we next calculated the difference between the basic and the acidic habitat. Finally, we evaluated if this B‐A difference in SNP density was related to the frequency of the acidic allele in the marine population. Our expectation was a negative relationship, indicating a particularly strong reduction in genetic diversity in the acidic populations at those core SNPs at which the acidic allele had to rise from particularly low initial frequency during adaptation. The random SNPs were again used analogously as a negative control. A robustness check for this analysis of selective sweeps is presented in Supporting Information Figure S8.

Unless specified otherwise, all analyses were performed with the R language (R Core Team 2017; codes for the main analyses are available on Dryad). Variation around estimated statistics was quantified through bootstrapping (Manly 2006) with 10,000 iterations.

## Results and Discussion

### BASIC AND ACIDIC STICKLEBACK ECOTYPES ON NORTH UIST HAVE EVOLVED INDEPENDENTLY

**Figure 2:**
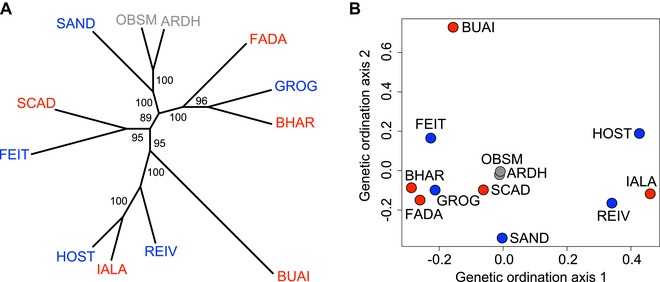
(A) Unrooted nuclear maximum likelihood tree based on 15,058 loner SNPs located in the high‐recombination chromosome peripheries and showing low AFD in between‐habitat population comparisons, using a single synthetic stickleback individual per population. Color coding is by habitat, as in Figure 1. The numbers give bootstrap support for all nodes. Additional trees based on multiple synthetic individuals per population, neighbor joining, the full genome‐wide set of loner SNPs, or just loner SNPs spaced by at least 1 Mb are shown in Supporting Information Figures S2, S9, S10, and S11). (B) Genetic similarity among the populations shown by their position along the first two NMDS ordination axes.

In our nuclear SNP phylogeny based on synthetic genotypes, basic and acidic populations appeared well‐mixed across the genealogical tree. Some terminal bifurcations, for instance, involved a basic and an acidic population originating from geographically distant lakes (HOST‐IALA, BHAR‐GROG) (Fig. 2A and Supporting Information Figs. S2, S9, S10, and S11). Conversely, populations from lakes located in closest geographic proximity and belonging to the same ecotype (GROG‐REIV and FADA‐IALA) appeared on distinct basal branches of the tree. Ordination of the populations also indicated the absence of genetic similarity by ecotype (Fig. 2B). (See also the weak correlations in allele frequencies estimated by BayPass for all population combinations except FEIT and GROG, Supporting Information Fig. S5B; these two populations may not qualify as fully independent.) These genetic patterns, combined with the geographic separation of the basic and acidic habitats due to surface geology, render the major alternative to parallel evolution—the single origin of a basic and an acidic ecotype followed by admixture between the ecotypes during secondary contact in different localities (Bierne et al. 2013)—highly implausible. Instead, our analyses support the view that our freshwater populations were founded independently by ancestral marine stickleback and then evolved in isolation from each other, consistent with the present‐day hydrological independence of the lakes (Fig. 1A) (further support for the conclusion of the evolutionary independence of our freshwater populations is elaborated in the Supporting Information “Discussion” section, paragraph 3). The multiple phenotypically similar populations within each lake type are thus well suited for an investigation of the genomics of parallel adaptation of an ancestral population to two ecologically distinct derived habitats.

### BASIC AND ACIDIC POPULATIONS HAVE DIVERGED IN PARALLEL IN NUMEROUS GENOMIC REGIONS

**Figure 3:**
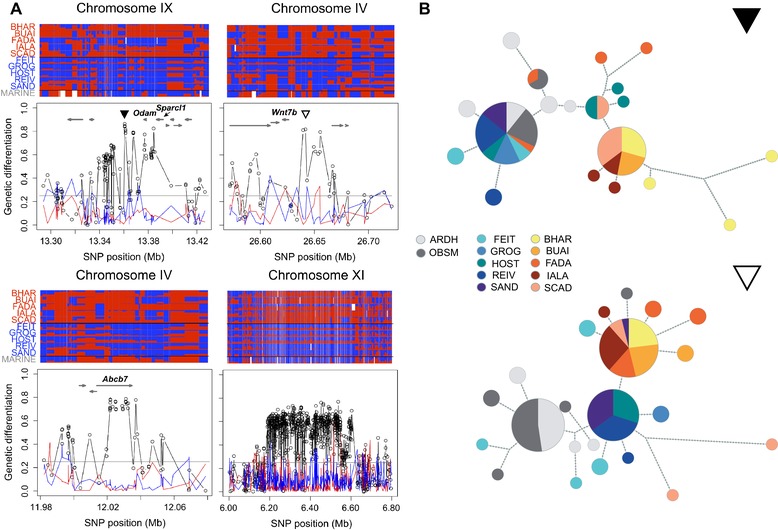
(A) Four exemplary genomic regions around core SNPs showing strong and highly parallel differentiation between basic and acidic stickleback ecotypes. The bottom panels show mean genetic differentiation (absolute allele frequency difference, AFD) profiles for the integrated B‐A (black), B‐B (blue), and A‐A (red) population comparisons. The dots represent individual SNPs, and the horizontal gray lines indicate genome‐wide median AFD for the integrated B‐A comparisons. Gray arrows show the location of genes (not for the large inversion on chromosome XI), with four candidates for B‐A adaptation labeled. The top panels summarize allele frequencies for each population at all SNPs underlying the AFD profiles on the bottom. Alleles are color coded in blue (basic alleles predominant in the basic ecotype pool) and red (acidic alleles). Cell widths are delimited by the midpoints between each focal SNP and its flanking SNPs. White cells represent missing data. (B) Haplotype genealogies based on SNPs from targeted individual‐level sequencing at the two top core SNPs. The position of the target segments is indicated by a filled (upper genealogy) and empty (lower genealogy) black triangle in (A). Each pie represents a unique haplotype (or a collection of closely related haplotypes, as these were collapsed; see “Methods” section), and edges connecting pies or nodes indicate one inferred mutational step.

Having obtained strong evidence that our focal freshwater stickleback populations adapted in parallel to basic and acidic lakes, our first main objective was to search for genomic regions playing a key role in the differentiation between basic and acidic ecotypes. After combining AFD data from all possible comparisons between basic and acidic lakes (B‐A comparisons), 42 independent genomic regions satisfied our criteria for loci under highly parallel B‐A differentiation. These regions generally contained multiple SNPs nearly fixed for alternative alleles between most populations from the two lake types (four examples are presented in Fig. 3A; the top [core SNP showing AFD > 0.75] 19 regions are visualized in detail in Supporting Information Fig. S12, and a genome‐wide differentiation plot is presented in Supporting Information Fig. S13). In these regions, we generally observed low differentiation *within* each habitat type (i.e., in the B‐B and A‐A comparisons; Fig. 3A and Supporting Information Fig. S12), indicating extensive haplotype sharing within each ecotype and hence ruling out the possibility that the populations adapted by selecting independent new mutations in these genomic regions (Roesti et al. 2014; Berner and Salzburger 2015). These conclusions—derived from pooled sequencing data—were supported by our individual‐level targeted sequencing at two top core SNPs: in both genomic regions, we observed that basic and acidic ecotypes formed distinct haplotype clusters, and that haplotypes identical by descent were shared among multiple populations within each ecotype (Fig. 3B).

A qualitative inspection of the 100 kb windows around the core SNPs suggested potential candidate genes for adaptive differentiation between basic and acidic environments. These included *Sparcl1* and *Odam* for the core SNP region on chromosome IX (Fig. 3A), both involved in vertebrate tissue mineralization and specifically bone and tooth development (Kawasaki et al. 2004; Kawasaki 2009). Other suggestive candidates were *Wnt7b* and *Abcb7* on chromosome IV (Fig. 3A). These latter genes have been suggested to be under divergent selection between marine and freshwater stickleback (Jones et al. 2012a; Jones et al. 2012b; Roesti et al. 2014; see also Supporting Information Fig. S3A), but here also appear involved in the differentiation between ecologically different *freshwater* habitats. A complete list of genes around the top core SNPs is presented as Supporting Information Table S4.

The core SNP regions also included an inversion of several hundred kilobases on chromosome XI (Fig. 3A), a locus commonly found highly differentiated between marine and freshwater stickleback (Hohenlohe et al. 2010; Jones et al. 2012b; Roesti et al. 2014; Supporting Information Fig. S3), but also between stickleback residing in adjoining lake and stream habitats exhibiting very similar water chemistry (Roesti et al. 2015). Divergent selection on this inversion across qualitatively different habitat transitions poses a major challenge to understanding what loci captured by the inversion are actually fitness‐relevant in each ecological context. The B‐A comparisons revealed two further genomic regions (on chromosomes V and XVII) showing extended population differentiation over hundreds of kilobases, although the consistency of differentiation across population comparisons was lower (Supporting Information Fig. S14). These regions further exhibited distinct MAF strata consistent with inversions (Roesti et al. 2015), but not the distortion in read alignment success characteristic of *ancient* inversion polymorphisms showing massive sequence differentiation (Roesti et al. 2013) (Supporting Information Fig. S14). We thus speculate that these regions may be relatively young inversions.

### BASIC‐ACIDIC DIFFERENTIATION THROUGH ASYMMETRIC SELECTION OF STANDING GENETIC VARIATION

**Figure 4:**
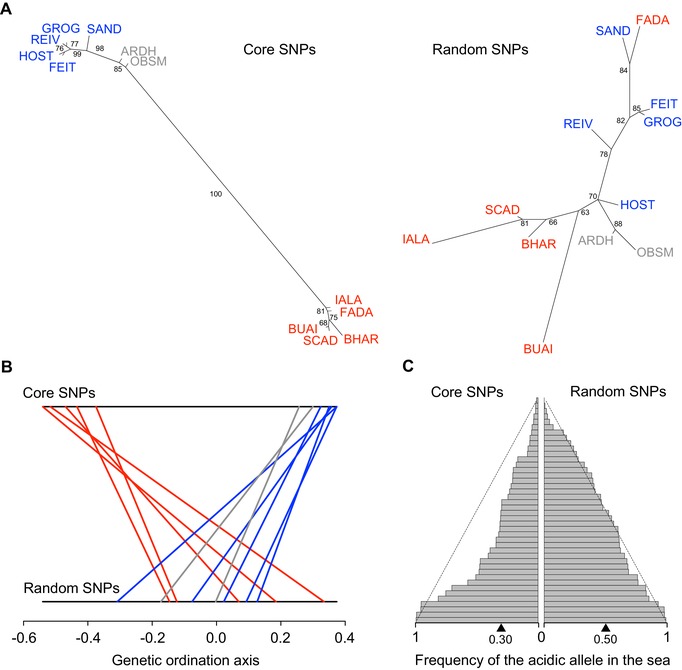
(A) Unrooted phylogenies based on one synthetic individual per population, generated by drawing alleles at random at the core SNPs representing 42 regions of strong basic‐acidic differentiation (left), and at 42 random SNPs (right). Population color codes follow Figure 1. Note the strong bootstrap support for the basal branches in the core SNP tree only. (B) Ordination (NMDS) of the populations, quantifying their genetic similarity across the 42 core (top) and random SNPs (bottom). Each line connects the position of a single population on the two coordinates. (C) Frequency distribution of the acidic allele in the marine stickleback across the core (left) and random SNPs (right). Within each category, the SNPs are shown in rows ordered by increasing frequency, and the black triangle on the bottom indicates their median frequency. The black dashed lines indicate allele frequencies expected under the uniform distribution.

The identification of genomic regions selected differentially between the two types of derived freshwater habitats motivated our second main prediction: that at loci of strong B‐A differentiation, the acidic ecotypes, residing in habitats more ecologically different from the ancestral marine habitat than the basic ecotypes, tend to have accumulated alleles uncommon in the ancestor. This prediction was confirmed by our phylogeny based on the core SNPs representing the 42 genomic regions of high B‐A differentiation (these core SNPs are characterized in detail in Supporting Information Table S5): in the genealogical tree, the basic populations formed a distinct branch clustering closely with the marine samples, whereas all acidic populations together formed a separate branch highly distinct from the one including the marine and basic fish (Fig. 4A left and Supporting Information Fig. S6A; see also Fig. 3B top for similar evidence based on individual‐level haplotype data). Consistent with the genome‐wide nuclear phylogeny (Fig. 2A), however, the genealogy based on the 42 random SNPs did not indicate a stronger genetic similarity of the basic than acidic ecotypes to the marine ancestor (Fig. 4A right). Likewise, our ordination analysis using the core SNPs revealed a close genetic similarity between basic and marine stickleback, with the acidic ecotypes appearing very different from these two (Fig. 4B top and Supporting Information Fig. S6B). By contrast, ordination based on the random SNPs indicated no genetic structure by habitat (Fig. 4B bottom).

These insights were refined by the inspection of allele frequencies within each population. Classifying the two alleles at the core SNPs as basic or acidic based on their overall frequency within the two freshwater habitats, we first observed that at these SNPs, the marine ancestor consistently harbored *both* alleles (Supporting Information Table S5). However, the acidic allele was the less common (i.e., the *minor* allele, frequency <0.5) in the marine fish at 32 of the 42 SNPs (two‐tailed binomial probability of an asymmetry of this magnitude or greater: *P* = 0.0009). The frequency distribution of the acidic alleles across the core SNPs was thus biased toward low values in marine stickleback and differed strikingly from uniformity expected for polymorphisms having segregated under selective neutrality for a long time (Wright 1931) (Fig. 4C left and Supporting Information Fig. S6C). The latter frequency distribution, however, was observed for the acidic alleles at the random SNPs (Fig. 4C right; the frequency distributions of the core and random SNPs differed clearly: *P* = 0.0083, two‐tailed permutation test with 9999 iterations using the absolute difference in the median frequencies as test statistic; Manly 2006).

Taken together, our analyses make clear that adaptive differentiation between basic and acidic stickleback ecotypes is built on the selection of genetic variation preexisting in the ancestor (i.e., standing genetic variation)—all core SNP alleles found in freshwater were also present in the sea. For the two core SNP regions scrutinized by targeted sequencing, the repeated use of standing variation during parallel evolution was confirmed directly by haplotype sharing among similar ecotypes from different lakes. Furthermore, adaptation to the ecologically relatively extreme acidic lakes has involved the accumulation of genetic variants relatively uncommon in marine fish, whereas the basic ecotypes have mostly retained the allele predominant in the sea. This B‐A asymmetry in marine core SNP allele frequencies provides a strong indication that these regions are truly involved in adaptation.

**Figure 5:**
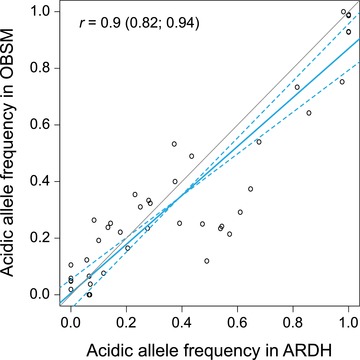
Association of the frequency of the acidic allele at the 42 core SNPs between the two marine stickleback samples (OBSM and ARDH). The slope of a major axis regression (blue line, with 95% confidence interval shown as dotted blue lines) is close to unity (gray line), indicating that the acidic alleles occur at similar frequencies in both samples. The regression statistic and associated 95% bootstrap CI are presented inside the graphic.

An intriguing question is why acidic core SNP alleles still occur at relatively appreciable frequencies in the sea (Fig. 4C left)—given that they represent polymorphisms tightly linked to genetic variants beneficial in an ecologically very different habitat type, and in part likely even coincide with such variants. A first possibility is that the acidic alleles are recessive and hence deleterious in the sea only when homozygous, thus impeding their complete elimination by selection (e.g., Cresko et al. 2004). The observed frequencies of most acidic core SNP alleles in the sea, however, seem too high for this scenario. Another explanation is that these frequencies reflect an antagonism between purifying selection in the marine population and gene flow from the acidic ecotypes (i.e., migration‐selection balance), maintained by continued hybridization between acidic and marine stickleback. At first sight, this scenario may appear plausible, as marine stickleback are reported to migrate into coastal lagoons and some freshwater lakes on North Uist during the breeding period. However, hybridization between acidic and marine stickleback seems extremely rare (A.D.C. MacColl, personal observation). Fortunately, our data allow a more direct evaluation of the above migration‐selection balance scenario: if gene flow between acidic and marine stickleback was common, we should find acidic alleles at higher frequency in our marine sample taken close to the drainages of the acidic lakes (OBSM on the east side of North Uist; Fig. 1A) than in the sample taken near the drainages of the basic lakes (ARDH, west side). Interestingly, this prediction is not upheld; the frequency of the acidic alleles at the core SNPs did not differ between the two marine samples separated by hundreds of kilometers of shoreline (Fig. 5). The frequency in the sea of alleles important to adaptive B‐A differentiation is therefore not substantially influenced in the short term by gene flow from freshwater ecotypes. Instead, these frequencies seem characteristic of marine stickleback around North Uist *in general*.

As a potential alternative explanation to migration‐selection balance, we consider that alleles selected to high frequency in acidic lakes may not be deleterious within the marine habitat when segregating at modest frequency—a prediction from models of local adaptation involving polygenic traits (Latta 1998; Le Corre and Kremer 2012)—thus preventing their complete elimination in the sea. However, we cannot rule out the possibility that we systematically overestimate the marine frequencies of the actual variants favored in the acidic lakes, given that the physical linkage between these variants and the corresponding acidic core SNPs may not be perfect. Evaluating these different ideas will benefit from individual‐level whole‐genome sequence data from freshwater and marine stickleback on and around North Uist, and from direct information on the phenotypic role and fitness consequences of acidic freshwater alleles in the marine habitat.

### THE RISE OF UNCOMMON ALLELES HAS CAUSED MORE NUMEROUS SELECTIVE SWEEPS IN THE ACIDIC ECOTYPES

**Figure 6:**
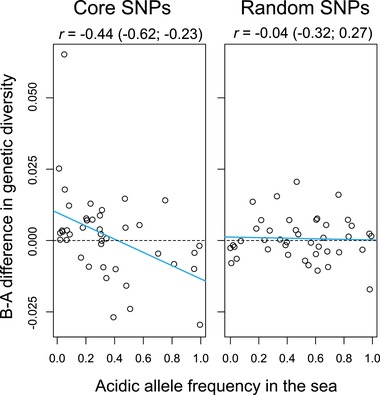
Selective sweeps in genomic regions important to B‐A differentiation. The difference in genetic diversity (relative density of high‐MAF SNPs) between basic and acidic populations across the 40 kb surrounding a focal SNP is plotted against the frequency of the acidic allele in the marine fish at the corresponding core (left) and random SNP (right). Each data point represents one of the 42 SNPs in each category. The statistics are Pearson's correlation coefficient along with its 95% bootstrap CI, and the blue lines show linear regressions (excluding the top left high‐residual core SNP had a minimal influence on the relationship: *r* = –0.45, 95% CI –0.66 to –0.18).

We have demonstrated that the differentiation between basic and acidic stickleback ecotypes on North Uist has generally involved the retention of alleles common in the ancestor in the basic lakes, and the selection of alleles uncommon in the ancestor in the acidic lakes. This implies that at loci important to B‐A differentiation, the basic populations must mostly have experienced relatively weak allele frequency changes, or no changes at all, whereas the acidic populations must have experienced stronger allele frequency changes and hence stronger associated reductions in genetic diversity (selective sweeps; Maynard Smith and Haigh et al. 1974; Kaplan et al. 1989; Hermisson and Pennings 2005; Messer and Petrov 2013). Our inspection of genetic diversity, quantified by the relative density of high‐MAF polymorphisms, across the 40 kb surrounding the core SNPs clearly supports this idea: the magnitude to which genetic diversity around a core SNP was reduced in acidic relative to basic stickleback was negatively correlated with the frequency of the corresponding acidic allele in the marine fish (Fig. 6 left). In other words, adaptation to the acidic lakes produced the strongest selective sweeps (positive B‐A difference in genetic diversity) around those acidic variants segregating at the lowest frequency in the sea. Conversely, around the few core SNPs at which the acidic allele was the *predominant* one in the sea, strong allele frequency changes and associated selective sweeps tended to occur within the *basic* populations (negative B‐A difference in genetic diversity). These observations offer a further validation of the ecological importance of the genomic regions tagged by our core SNPs. In addition, this analysis indicates that allele frequencies observed in present‐day marine stickleback must be similar enough to those in the true marine ancestor of our freshwater populations to still allow detecting their association with patterns of genetic diversity shaped during adaptation.

**Figure 7:**
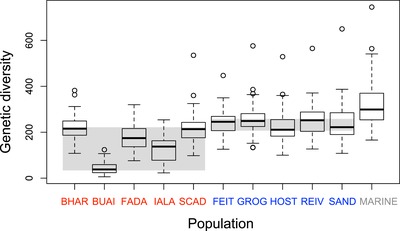
Genetic diversity within populations, expressed as the number of SNPs passing a MAF threshold of 0.3 across 1 Mb windows centered at the 42 random SNPs, visualized by standard boxplots (i.e., thick lines represent the medians, rectangular boxes the interquartile ranges, IQR). The populations are ordered by habitat (acidic, basic, and marine). The gray background rectangles indicate the range of the medians across all five populations within the basic and within the acidic ecotypes. The marine population reflects the combination of the OBSM and ARDH samples; considering the larger of the two (OBSM, *N* = 20) alone, however, leads to very similar results (OBSM only: median diversity = 294, IQR = 251–356; OBSM and ARDH combined: median = 299, IQR = 254–370).

At the random SNPs, we found no clear relationship between the frequency of the acidic alleles in the sea and habitat‐related bias in genetic diversity (Fig. 6 right), as expected for polymorphisms neutral to B‐A ecology. However, inspecting genetic diversity across an extended (1 Mb) segment around the random SNPs revealed interesting habitat‐related patterns: the acidic populations tended to harbor lower diversity than the basic ones (two‐tailed permutation test using population medians as data points and the B‐A median difference as test statistic: *P* = 0.0454), and the highest diversity occurred in the marine fish (Fig. 7). The latter observation conforms to the common trend of marine stickleback to exhibit large effective population sizes—allowing the maintenance of elevated genetic diversity—relative to derived freshwater populations (Mäkinen et al. 2006; Hohenlohe et al. 2010; Catchen et al. 2013). The finding of low genetic diversity in the acidic ecotypes, however, seems surprising: the three largest lakes in our study are acidic (Fig. 1A), which would lead to the expectation of larger effective population sizes in the acidic than the basic lakes on average, and hence relatively reduced genetic diversity in the latter. However, our environmental and phenotypic data and our analyses of core SNP alleles consistently indicate that acidic lakes are ecologically more different from the ancestral habitat than the basic lakes. Ancestral colonizers must therefore have been exposed to particularly intense selection (i.e., been more strongly maladapted) within the acidic lakes, implying an initial period of particularly low population density. Moreover, acidic lakes display lower productivity than basic lakes (Waterston et al. 1979) and may therefore support relatively reduced population densities even in the long term. Both of these conditions would have promoted the stochastic loss of genetic variation in the acidic ecotypes. Clearly, our derived freshwater populations, and especially the acidic ones, lost genetic diversity not only within localized regions around targets of selection, but also genome‐wide due to habitat‐related differences in effective population size.

## Conclusions

Our study shows that the emergence of similar ecotypes within multiple derived habitats can result in parallel evolution at the genomic level. We further demonstrate how insights into the differentiation of derived populations can be strengthened by including genetic data from their recent common ancestor: in our stickleback system, basic‐acidic differentiation occurred via the genome‐wide sorting of standing variation in the ancestor, and asymmetry in this sorting is predictable from the difference of each derived habitat from the ancestral one. The detection of numerous genomic regions repeatedly involved in basic‐acidic differentiation now provides a resource for identifying the associated phenotypes and exploring their ecological function. Such work may reveal whether genomic regions showing the strongest parallelism include developmental components of bony armor traits, or if they represent more elusive aspects of adaptive differentiation between basic and acidic waters.

## CONFLICT OF INTEREST

The authors declare no conflicts of interest.

Associate Editor: Z. Gompert

## Supporting information


**Method**.
**Discussion**.
**Table S1**. Description of study habitats and populations.
**Table S2**. All pairwise populations comparisons (AFD and F_*ST*_).
**Table S3**. Pairwise comparisons per habitat (AFD and F_*ST*_).
**Table S4**. List of genes around the top core SNPs.
**Table S5**. Characterization of the 42 core SNPs.
**Figure S1**. Schematic description of the SNP generation protocol.
**Figure S2**. Unrooted nuclear phylogeny (neutral SNPs).
**Figure S3**. Marine‐freshwater differentiation by chromosome.
**Figure S4**. Standardized basic‐acidic differentiation.
**Figure S5**. BayPass analysis.
**Figure S6**. Robustness check for random SNPs.
**Figure S7**. Comparision SNP density vs. nucleotide diversity (π).
**Figure S8**. Robustness check for selective sweeps.
**Figure S9**. Unrooted nuclear phylogeny (NJ tree, neutral SNPs).
**Figure S10**. Unrooted nuclear phylogeny (all loner SNPs).
**Figure S11**. Unrooted nuclear phylogeny (SNP spacing at least 1Mb).
**Figure S12**. Description of the top core SNPs.
**Figure S13**. Genome‐wide basic‐acidic differentiation.
**Figure S14**. Potential chromosomal inversions.
