# Supplementary material for: Predictable genome‐wide sorting of standing genetic variation during parallel adaptation to basic versus acidic environments in stickleback fish
Source: Evol Lett. 2019 Jan 23;3(1):28–42. doi: 10.1002/evl3.99 (PMC6369934; doi:10.1002/evl3.99)
Supplement: Supplementary file 1 — Method. Discussion. Table S1. Description of study habitats and populations. Table S2. All pairwise populations comparisons (AFD and FST). Table S3. Pairwise comparisons per habitat (AFD and FST). Table S4. List of genes around the top core SNPs. Table S5. Characterization of the 42 core SNPs. Figure S1. Schematic description of the SNP generation protocol. Figure S2. Unrooted nuclear phylogeny (neutral SNPs). Figure S3. Marine‐freshwater differentiation by chromosome. Figure S4. Standardized basic‐acidic differentiation. Figure S5. BayPass analysis. Figure S6. Robustness check for random SNPs. Figure S7. Comparision SNP density vs. nucleotide diversity (π). Figure S8. Robustness check for selective sweeps. Figure S9. Unrooted nuclear phylogeny (NJ tree, neutral SNPs). Figure S10. Unrooted nuclear phylogeny (all loner SNPs). Figure S11. Unrooted nuclear phylogeny (SNP spacing at least 1Mb). Figure S12. Description of the top core SNPs. Figure S13. Genome‐wide basic‐acidic differentiation. Figure S14. Potential chromosomal inversions. [file EVL3-3-28-s001.pdf]

Supporting Information

Predictable genome-wide sorting of standing genetic variation during parallel adaptation to basic versus acidic environments in stickleback fish

Quiterie Haenel[1], Marius Roesti[1,2,3], Dario Moser[1,4], Andrew D. C. MacColl[5] and Daniel Berner[1]

[1] Department of Environmental Sciences, Zoology, University of Basel, 4051 Basel, Switzerland

[2] Biodiversity Research Centre and Zoology Department, University of British Columbia, Vancouver, British Columbia V6T 1Z4, Canada

[3] *Current address*: Institute of Ecology and Evolution, University of Bern, 3012 Bern, Switzerland

[4] *Current address*: Jagd- und Fischereiverwaltung Thurgau, 8510 Frauenfeld, Switzerland

[5] School of Life Sciences, University of Nottingham, Nottingham NG7 2RD, United Kingdom

Corresponding authors:

- Quiterie Haenel: quiterie.haenel@unibas.ch
- Daniel Berner: daniel.berner@unibas.ch

Contents

Methods……………………………………..........................………..…..Pages 3-4

Discussion…………………….................……………......……………Pages 5-10

Tables…………………...............……………………………..........…...Pages 11-31

Table S1: Description of study habitats and populations...............................Pages 11-12

Table S2: All pairwise populations comparisons (AFD and F_{ST})..................Page 13

Table S3: Pairwise comparisons per habitat (AFD and F_{ST})..........................Page 14

Table S4: List of genes around top core SNPs..Pages 15-26

Table S5: Characterization of the 42 core SNPs..Pages 27-31

Figures………………………………………….……….……….…...Pages 32-57

Figure S1: Schematic description of the SNP generation protocol...............Page 32

Figure S2: Unrooted nuclear phylogeny (neutral SNPs).................................Page 33

Figure S3 Marine-freshwater differentiation by chromosome.........................Pages 34-36

Figure S4: Standardized basic-acidic differentiation...Pages 37-38

Figure S5: BayPass analysis...Pages 39-40

Figure S6: Robustness check for random SNPs..Page 41

Figure S7: Comparison SNP density vs. nucleotide diversity (π)..................Pages 42-44

Figure S8: Robustness check for selective sweeps..Page 45

Figure S9: Unrooted nuclear phylogeny (NJ tree, neutral SNPs).................Page 46

Figure S10: Unrooted nuclear phylogeny (all loner SNPs)..............................Page 47

Figure S11: Unrooted nuclear phylogeny (SNP spacing at least 1Mb).......Page 48

Figure S12: Description of the top core SNPs...Pages 49-54

Figure S13: Genome-wide basic-acidic differentiation......................................Page 55

Figure S14: Potential chromosomal inversions...Pages 56-57

References……………………………...…………………….…..…...Pages 58-59

Methods

Targeted individual-level Sanger sequencing was performed at two top AFD extremes by amplifying a 700 bp fragment from a subsample of 4-8 individuals from each of the 12 populations. Primer pairs and PCR conditions were as follows:

Chromosome IX, SNP position (bp): 13,360,688

Primer	Sequence
Forward	5'CAGTCAGAGGACCGGACGT3'
Reverse	5'ATCTCTGCTGATGGTTGGCA3'

For a 12.5uL reaction volume, we used 1.25uL Taq polymerase buffer (x10), 1uL dNTP mix (final concentration of each dNTP 200uL), 0.25uL of each primer at 10uL, 1uL of DNA template, 0.50uL of Red Taq DNA polymerase and 8.25uL of sterile deionized water. Cycling conditions were 2 min at 94°C (1 cycle); 30 sec at 94°C, 30 sec at 60°C, 1 min at 72°C (30 cycles); 7 min at 72°C (1 cycle). PCR success was confirmed on a 1.5% agarose gel.

Chromosome IV, SNP position (bp): 26,641,811

Primer	Sequence
Forward	5'AGCCACAATGCCAAAGGACA3'
Reverse	5'CAAATCCAAACACTCGGGTGG3'

For a 12.5uL reaction volume, we used 1.25uL Taq polymerase buffer (x10), 1uL dNTP mix (final concentration of each dNTP 200uL), 0.25uL of each primer at 10uL,

1uL of DNA template, 0.50uL of Red Taq DNA polymerase, 0.75uL of $MgCl_2$ and 7.5uL of sterile deionized water. Cycling conditions were 2 min at 94°C (1 cycle); 30 sec at 94°C, 30 sec at 54°C, 1 min at 72°C (30 cycles); 7 min at 72°C (1 cycle). PCR success was confirmed on a 1.5% agarose gel.

Discussion

This discussion presents additional detail and evidence supporting conclusions drawn in the main paper.

1) Is it valid to combine the two marine samples to a single marine population in the present study? Would exploring the evolutionary independence of the derived freshwater populations not require including samples from additional marine populations?

Very generally, marine stickleback occurring in a broader geographic region are considered a large, genetically well mixed population; they display very limited genetic structure compared to derived freshwater populations from the same region, and elevated genetic diversity relative to freshwater populations (Hohenlohe et al. 2010; Jones et al. 2012a; Catchen et al. 2013; Roesti et al. 2014). As expected, these classical genetic patterns are also observed in the present study: although our two marine samples were taken from sites separated by more than a hundred kilometers of shoreline (Fig. 1A), their comparison yields a median genome- wide differentiation of only 0.07 (AFD) and 0.01 (F_{ST}) (Table S2 and S3; note that this magnitude of genetic differentiation is almost certainly overestimated because the marine samples were substantially smaller [N = 10 and 20] than all the freshwater samples [Table S1], and the associated imprecision in allele frequency estimation should bias both median AFD and F_{ST} upward). By contrast, median genome-wide differentiation averaged across all comparisons within each freshwater habitat type (both N = 10) is much greater (basic-basic comparisons: AFD = 0.17, F_{ST} = 0.04; acidic-acid comparisons: AFD = 0.25, F_{ST} = 0.09), despite the populations within

each freshwater habitat type being separated by much smaller geographic distances. Moreover, Fig. 7 reveals greater genetic diversity in the marine than the freshwater fish, consistent with the generally large effective population size of marine stickleback.

While these observed patterns of genetic differentiation and diversity are fully consistent with work on marine and freshwater stickleback worldwide and indicate that marine stickleback around North Uist can be considered a large, well-mixed population, even more compelling evidence emerges from our phylogenies: in all trees (Fig. 2A, Fig S2, S9, S10, S11), the branches connecting each marine sample (OBSM, ARDH) to their first common node are very similar in length. This means that no marine sample can be considered closer to any of the freshwater populations than the other marine sample (a similar pattern emerges when exploring population similarity by ordination, Fig. 2B). This in turn implies that even a single marine sample would provide a sufficient proxy of the marine ancestor of all the derived freshwater populations. Clearly, combining our two marine samples to a single biological population is a valid approach; additional marine samples are not needed for our analyses.

2) The generation of synthetic individuals based on pooled sequencing genotype data generates artificial linkage equilibrium among alleles – could this bias phylogenetic inference in the present study?

As a robustness check of using synthetic individuals for phylogenetic inference, we repeated the phylogenetic analysis using individuals generated by concatenating nucleotides from SNPs spaced by a minimum of 1 Mb only. Since linkage

disequilibrium has been observed in threespine stickleback to decay over a physical distance of a few kilobases (e.g., Roesti et al. 2015), this spacing should ensure that concatenated alleles can also occur on the same DNA molecule in nature. Despite limited marker resolution (227 SNPs only), this alternative phylogenetic analysis (Fig. S11) recovered the main features of the high-resolution trees.

We recognize, however, that the concatenation of nucleotides from a pool may be problematic when linkage disequilibrium within populations is strong over large physical scales. The main scenario able to generate such linkage disequilibrium is recent dispersal among populations. In this case, long immigrant haplotype tracts differing from the standard genetic composition of a given population would be disintegrated during DNA pooling so that synthetic individuals derived from the pooled sequence data would appear more similar in the phylogenetic tree than would real individual genotypes. A scenario of recent dispersal among populations, however, can be ruled out for our study: first, all our populations show strong pair-wise genetic differentiation from each other (Table S2 and S3; see also previous paragraph). Second, given the present-day hydrology of the study system, only marine-freshwater dispersal would be plausible. However, marine fish are phenotypically distinct from the basic and acidic ecotypes, so that marine-freshwater migrants (and likely even recent hybrids and backcrosses) could be identified phenotypically. Our phenotypic analysis, however, yielded no indication of migration or hybridization. Third, a recent study using individual-level sequence data (Magalhaes et al. 2016), covering seven out of our ten freshwater populations, found no indication of population admixture (Fig. 3 in that paper). We therefore see no reason to assume long-range linkage disequilibrium within our populations, and are

confident that our phylogenetic analysis using nucleotide concatenation produces reliable insights into the genetic similarity among our study populations. In support of this view, our general observation of population monophyly is consistent with monophyly observed in a tree based on individual-level genotype data from a subset of our study populations (Fig. S1 in Magalhaes et al. 2016).

3) Can the study rule out the possibility that each of the two freshwater stickleback ecotypes (basic and acidic) evolved only once on North Uist, expanded geographically, came into secondary contact, and started hybridizing? The resulting genetic exchange may have caused some basic and acidic populations to cluster together on the terminal branches of the genealogical tree, thus falsely suggesting the repeated independent differentiation of basic and acidic populations (Bierne et al. 2013).

This possibility appears extremely unparsimonious when interpreting our phylogenetic trees (Fig. 2A, S2 and S9, S10, S11) in the light of the geographic arrangement of the lakes and habitat types. Specifically, because of the geologically determined (Waterson et al. 1979; Giles 1983) spatial segregation between the two habitat types (basic in the west and acidic in the east; Fig. 1A), it does not appear physically and ecologically plausible that an ancient acidic ecotype dispersed to the basic region and vice versa. The basic and acidic catchments are widely separated in space, and the only aquatic route between them is through the sea. In addition, the specific habitat appropriate to each ecotype would have been missing in the newly invaded region, making successful dispersal highly unlikely. Secondary contact and introgressive genetic exchange between the ecotypes across the entire island is

therefore not realistic for geological and ecological reasons. The relative genetic similarity between, for example, the HOST and IALA populations (observed consistently in all our genealogies, see Fig. 2A, S2 and S9, S10, S11) can only be explained plausibly by *independent* colonization from a large, genetically well-mixed marine population, followed by the stochastic sorting of ancestral neutral variation that has resulted in these populations being relatively similar genetically by chance. Moreover, if dispersal and gene flow had been extensive at the scale of the entire island, it would be hard to explain why populations of the *same* ecotype and residing in close geographic neighborhood (e.g., the IALA and BUAI populations) consistently emerge as genetically distant in all our phylogenies, and also in the ordination (Fig. 2B). The repeated stochastic sorting of neutral genetic variation from a shared marine ancestor during independent evolution is the only explanation parsimoniously reconciling our tree topology with the geography of the study populations. Our tree- and ordination-based inference of evolutionary independence is also supported by the general absence of substantial allele frequency correlation between populations, as estimated by BayPass (Fig. S5B), and fully consistent with our study lakes currently draining independently into the sea (not confirmed for FEIT; Fig. 1A).

Further evidence of the repeated, independent evolution of similar ecotypes in multiple lakes derives from the non-perfect phenotypic parallelism among the acidic populations (Fig. 1B, Table S1): the IALA population, for instance, exhibits a fully developed pelvic structure like the basic ecotype, whereas the FADA population in very close neighborhood (Fig. 1A) has completely lost its pelvic structure. Such genetically based phenotypic differences are difficult to explain when assuming the formation and spread of a single ancestral acidic ecotype across the acidic side of

North Uist. Conversely, the relative similarity of the basic populations (Fig. 1B, Table S1) does not imply that they derive from a single ancestral basic ecotype that emerged once on North Uist; the basic populations correspond phenotypically to the standard freshwater stickleback ecotype known to have evolved independently through parallel differentiation from marine ancestors countless times all across the species' range (Bell & Foster 1994).

Tables

Table S1: Characterization of the lakes and lagoons from which the stickleback samples were collected, and number of individuals sampled from each site (numbers in parentheses indicate sample sizes underlying Sanger sequencing of each of the two loci in Fig. 3). Data on pH, water surface, and calcium concentration (Ca2+) and lake surface are from Magalhaes *et al.* (2016); this publication also provides geographic coordinates of all lakes. Armor trait data are averaged over 20 individuals chosen at random within each sample (with the exception of the marine samples that were considered in full). Lateral plate number refers to a single body side.

**Habitat type**	**Site code**	**Site name**	**N**	**pH**	**[Ca^{2+}] (10^{-5} mg/L)**	**Water surface (ha)**	**Lateral plate number**	**Dorsal spines number**	**Presence of a pelvic complex (%)**
Acidic	BHAR	a' Bharpa	30 (4/3)	6	3.42	53.9	0	1.85	0
Acidic	BUAI	na Buaile	30 (4/4)	6.7	6.01	1.7	2.95	3	30
Acidic	FADA	Fada	23 (4/4)	6.7	4.06	160.0	0	0.5	0
Acidic	IALA	Ialaidh	26 (4/4)	6.4	5.95	0.4	3.05	2.45	100
Acidic	SCAD	Scadavary	30 (4/2)	6.1	3.27	551.6	0.2	1.65	5
Basic	FEIT	nam Feithean	30 (3/4)	8.3	77.6	15.7	4.25	2.95	100
Basic	GROG	Grogary	30	8.2	63.8	14.8	2.95	2.95	100

			(4/3)						
Basic	HOST	Hosta	30 (4/4)	8.3	72.3	25.8	3.4	3	100
Basic	REIV	na Reival	29 (4/4)	9	44.9	6.1	3.4	3	100
Basic	SAND	Sandary	30 (3/4)	8.3	75.2	15.5	3	3	100
Marine	ARDH	Ard Heisker	10 (7/8)	8.6	498.5	-	25	3	100
Marine	OBSM	Ob’ nan Stearnain	20 (7/8)	9.1	487.1	-	25	3	100

Table S2: Genome-wide mean (lower-left semimatrix) and median (upper-right semimatrix) genetic differentiation, expressed as absolute allele frequency difference (AFD), and as F_{ST} (Nei's 1973 estimator G_{ST}) in parentheses, for all pairwise populations comparisons.

	BHAR	BUAI	FADA	IALA	SCAD	FEIT	GROG	HOST	REIV	SAND	ARDH	OBSM
BHAR		0.344 (0.171)	0.219 (0.068)	0.246 (0.086)	0.160 (0.038)	0.207 (0.055)	0.218 (0.061)	0.214 (0.061)	0.210 (0.061)	0.205 (0.057)	0.196 (0.066)	0.192 (0.063)
BUAI	0.384 (0.252)		0.339 (0.169)	0.287 (0.132)	0.349 (0.175)	0.363 (0.184)	0.387 (0.202)	0.376 (0.195)	0.368 (0.192)	0.358 (0.182)	0.345 (0.204)	0.354 (0.211)
FADA	0.267 (0.133)	0.395 (0.275)		0.246 (0.088)	0.215 (0.068)	0.238 (0.076)	0.241 (0.078)	0.238 (0.077)	0.242 (0.082)	0.224 (0.071)	0.226 (0.089)	0.227 (0.086)
IALA	0.302 (0.164)	0.385 (0.277)	0.314 (0.184)		0.249 (0.088)	0.267 (0.096)	0.272 (0.099)	0.262 (0.094)	0.261 (0.097)	0.245 (0.086)	0.247 (0.105)	0.250 (0.107)
SCAD	0.206 (0.085)	0.387 (0.257)	0.264 (0.131)	0.304 (0.167)		0.211 (0.058)	0.219 (0.063)	0.217 (0.063)	0.219 (0.066)	0.200 (0.056)	0.200 (0.068)	0.195 (0.065)
FEIT	0.249 (0.109)	0.386 (0.240)	0.281 (0.138)	0.306 (0.159)	0.252 (0.112)		0.074 (0.008)	0.139 (0.027)	0.184 (0.045)	0.201 (0.052)	0.194 (0.059)	0.191 (0.056)
GROG	0.262 (0.119)	0.412 (0.266)	0.287 (0.146)	0.314 (0.169)	0.263 (0.122)	0.090 (0.017)		0.135 (0.026)	0.192 (0.050)	0.207 (0.056)	0.207 (0.068)	0.205 (0.066)
HOST	0.261 (0.122)	0.406 (0.268)	0.289 (0.149)	0.311 (0.170)	0.265 (0.127)	0.176 (0.061)	0.171 (0.058)		0.200 (0.054)	0.210 (0.059)	0.197 (0.064)	0.194 (0.061)
REIV	0.256 (0.120)	0.400 (0.267)	0.291 (0.153)	0.312 (0.173)	0.267 (0.131)	0.223 (0.092)	0.234 (0.101)	0.244 (0.110)		0.212 (0.062)	0.176 (0.054)	0.177 (0.054)
SAND	0.255 (0.119)	0.394 (0.261)	0.277 (0.143)	0.296 (0.159)	0.251 (0.119)	0.242 (0.106)	0.252 (0.114)	0.257 (0.120)	0.259 (0.123)		0.196 (0.067)	0.194 (0.064)
ARDH	0.247 (0.150)	0.382 (0.282)	0.275 (0.171)	0.297 (0.189)	0.250 (0.152)	0.236 (0.133)	0.250 (0.148)	0.244 (0.143)	0.223 (0.132)	0.244 (0.150)		0.073 (0.010)
OBSM	0.243 (0.143)	0.390 (0.286)	0.274 (0.166)	0.300 (0.187)	0.247 (0.147)	0.233 (0.128)	0.247 (0.143)	0.241 (0.137)	0.224 (0.129)	0.242 (0.143)	0.096 (0.023)	

Table S3: Genome-wide mean and median AFD and F_{ST} (Nei's 1973 estimator G_{ST}) averaged across all population comparisons available for each habitat comparison category (A = acidic, B = basic, M = marine; number of population comparisons in parentheses).

	**Mean**		**Median**	
**Habitats**	**AFD**	**F_{ST}**	**AFD**	**F_{ST}**
A vs. A (10)	0.319	0.191	0.252	0.094
B vs. B (10)	0.216	0.091	0.165	0.038
B vs. A (25)	0.300	0.161	0.250	0.087
M vs. A (10)	0.289	0.185	0.234	0.097
M vs. B (10)	0.238	1.137	0.192	0.060
M vs. FW (20)	0.262	0.160	0.210	0.075
M vs. M (1)	0.096	0.023	0.073	0.010

Table S4: List of all the genes present in a 100 kb window around 19 core SNPs passing the stringent AFD threshold of 0.75 (i.e., the genome regions A to S characterized in Fig. S8).

ID	Chr	N°	Gene ID	Name	Start position	End position	Description
**A**	chrIX	1	ENSGACG00000017898	*odam*	13376375	13378298	odontogenic, ameloblast asssociated
		2	ENSGACG00000017900	*CNGA1*	13331902	13335265	cyclic nucleotide gated channel alpha 1
		3	ENSGACG00000017892	*sparcl1*	13386748	13392238	SPARC-like 1
		4	ENSGACG00000017889		13394935	13397012	osteopontin domain
		5	ENSGACG00000017903	*TACR3*	13314220	13326105	tachykinin receptor 3
		6	ENSGACG00000017887	*aptx*	13398416	13400992	aprataxin
		7	ENSGACG00000017879	*dnaja1*	13401116	13407421	DnaJ (Hsp40) homolog, subfamily A, member 1
		8	ENSGACG00000017872	*smu1b*	13410460	13415580	smu-1 suppressor of mec-8 and unc-52 homolog b (C. elegans)

**B**	chrI	1	ENSGACG00000004934	*supt5h*	866290	879634	SPT5 homolog, DSIF elongation factor subunit
		2	ENSGACG00000004963	*cox7a1*	885557	886553	cytochrome c oxidase subunit VIIa polypeptide 1 (muscle)
		3	ENSGACG00000004964	*nf1a*	889314	922850	neurofibromin 1a
		4	ENSGACG00000004929	*triap1*	864993	865217	TP53 regulated inhibitor of apoptosis 1
		5	ENSGACG00000004927		846012	847417	
		6	ENSGACG00000004922		835507	842787	
		7	ENSGACG00000004992	*smco4*	925191	925370	single-pass membrane protein with coiled-coil domains 4
**C**	chrIV	1	ENSGACG00000018959	*wnt7b*	26620620	26626229	wingless-type MMTV integration site family, member 7Ba
		2	ENSGACG00000018960	*atxn10*	26609883	26617247	ataxin 10
		3	ENSGACG00000018958	*pparaa*	26666495	26676255	peroxisome proliferator-activated receptor alpha a

		4	ENSGACG00000018964	*FBLN1*	26571948	26609189	fibulin 1
		5	ENSGACG00000018957	*si:ch211-239e6.4*	26680344	26681228	cysteine rich DPF motif domain containing 1
**D**	chrVII	1	ENSGACG00000020121	*lim2.1*	13813116	13815236	lens intrinsic membrane protein 2.1
		2	ENSGACG00000020120	*bsx*	13808290	13809957	brain-specific homeobox
		3	ENSGACG00000020119		13803433	13805401	
		4	ENSGACG00000020118		13799154	13803100	
		5	ENSGACG00000020117	*hspa8*	13782601	13786688	heat shock protein 8
		6	ENSGACG00000020116		13776594	13781629	
**E**	chrXX	1	ENSGACG00000007563		10619866	10623759	
		2	ENSGACG00000007569		10613890	10618424	
		3	ENSGACG00000007594	*zgc:171592*	10611768	10613631	chymotrypsin-like
		4	ENSGACG00000007597	*si:dkey-*	10607579	10608562	

				117a8.4			
		5	ENSGACG00000007546	*si:ch73-380l3.1*	10634711	10725421	
		6	ENSGACG00000007600	*lin37*	10602974	10605770	lin-37 DREAM MuvB core complex component
		7	ENSGACG00000007557		10637722	10708611	
		8	ENSGACG00000007618		10598096	10599752	
		9	ENSGACG00000007622	*hspb6*	10596751	10597728	heat shock protein, alpha-crystallin-related, b6
		10	ENSGACG00000007626	*psenen*	10593675	10594992	presenilin enhancer gamma secretase subunit
		11	ENSGACG00000007639		10573314	10585770	
		12	ENSGACG00000007659	*igflr1*	10565975	10570695	IGF-like family receptor 1
**F**	chrV	1	ENSGACG00000005578	*pemt*	3912342	3951075	phosphatidylethanolamine N-methyltransferase
		2	ENSGACG00000005572	*rasd1*	3956364	3957372	RAS, dexamethasone-induced 1
		3	ENSGACG00000005546	*NT5C*	3982133	3987560	5', 3'-nucleotidase, cytosolic

		4	ENSGACG00000005506	*cops3*	3987270	3995324	COP9 signalosome subunit 3
		5	ENSGACG00000005496	*usp22*	3996761	4006817	ubiquitin specific peptidase 22
**G**	chrIV	1	ENSGACG00000018803	*rassf8b*	28641481	28646130	Ras association (RalGDS/AF-6) domain family (N-terminal) member 8b
**H**	chrIV	1	ENSGACG00000018231	*abcb7*	12013745	12034920	ATP-binding cassette, sub-family B (MDR/TAP), member 7
		2	ENSGACG00000018229	*uprt*	12009666	12013671	uracil phosphoribosyltransferase (FUR1) homolog (S. cerevisiae)
		3	ENSGACG00000018224	*zdhhc15b*	12003229	12006996	zinc finger, DHHC-type containing 15b
**I**	chrXIV	1	ENSGACG00000017111	*pptc7b*	7257631	7262371	PTC7 protein phosphatase homolog b
		2	ENSGACG00000017108	*prnpb*	7255200	7256612	prion protein b
		3	ENSGACG00000017119	*aplnrb*	7265980	7266984	apelin receptor b
		4	ENSGACG00000017107		7252229	7252705	
		5	ENSGACG00000017120		7268685	7272968	

		6	ENSGACG00000017101	*kcnip3b*	7241177	7250465	Kv channel interacting protein 3b, calsenilin
		7	ENSGACG00000017093	*trim69*	7234209	7237347	tripartite motif containing 69
		8	ENSGACG00000017091	*bmp1b*	7223141	7233079	bone morphogenetic protein 1b
		9	ENSGACG00000017126	*nfkbil1*	7290042	7291957	nuclear factor of kappa light polypeptide gene enhancer in B-cells inhibitor-like 1
		10	ENSGACG00000017132	*atp6v0a2a*	7293761	7300431	ATPase, H+ transporting, lysosomal V0 subunit a2a
		11	ENSGACG00000017088	*antxr1b*	7212387	7221007	anthrax toxin receptor 1b
		12	ENSGACG00000017144	*osbp2*	7302479	7311793	oxysterol binding protein 2
**J**	chrXVII	1	ENSGACG00000007398	*foxj3*	6755976	6790998	forkhead box J3
		2	ENSGACG00000007391		6746613	6750817	
		3	ENSGACG00000007405	*ppcs*	6818104	6820519	phosphopantothenoylcysteine synthetase
		4	ENSGACG00000007417	*utp3*	6819603	6822503	UTP3, small subunit (SSU) processome component, homolog (S. cerevisiae)

		5	ENSGACG00000007358	*syn2b*	6695094	6740553	synapsin IIb
		6	ENSGACG00000007429		6824354	6825105	
		7	ENSGACG00000007430		6826808	6829333	
		8	ENSGACG00000007437	*si:dkey-264d12.4*	6830166	6831109	epithelial membrane protein 3
		9	ENSGACG00000007365	*TIMP4*	6726433	6733199	TIMP metallopeptidase inhibitor 4
**K**	chrVII	1	ENSGACG00000020350	*rtn2b*	19983607	19986866	reticulon 2b
		2	ENSGACG00000020349	*ppm1nb*	19977504	19981930	protein phosphatase, Mg2+/Mn2+ dependent, 1Nb (putative)
		3	ENSGACG00000020348	*kcnk12l*	19973787	19975812	potassium channel, subfamily K, member 12 like
		4	ENSGACG00000020351	*pvrl3b*	19998501	20009445	poliovirus receptor-related 3b
		5	ENSGACG00000020347	*itpkca*	19968056	19972062	inositol-trisphosphate 3-kinase Ca
		6	ENSGACG00000020346	*ccdc61*	19964556	19968807	coiled-coil domain containing 61

		7	ENSGACG00000020345		19945027	19963823	
		8	ENSGACG00000020352		20013617	20014501	
		9	ENSGACG00000020353	*ppme1*	20017457	20022592	protein phosphatase methylesterase 1
		10	ENSGACG00000020354	*ucp2*	20026028	20032135	uncoupling protein 2
		11	ENSGACG00000020344	*pls3*	19930922	19943010	plastin 3 (T isoform)
		12	ENSGACG00000020355	*dnajb13*	20031874	20034052	DnaJ (Hsp40) homolog, subfamily B, member 13
		13	ENSGACG00000020356	*rab6a*	20035866	20042328	RAB6A, member RAS oncogene family
**L**	chrXV	1	ENSGACG00000013078		16218640	16219585	
		2	ENSGACG00000013081	*vrk1*	16187758	16194595	vaccinia related kinase 1
		3	ENSGACG00000013067	*ak7b*	16254902	16265808	adenylate kinase 7b
**M**	chrXVII	1	ENSGACG00000007138	*atp2b2*	6457686	6506154	ATPase, Ca++ transporting, plasma membrane 2

		2	ENSGACG00000007204	*slc6a11b*	6519657	6535112	solute carrier family 6
**N**	chrXI	1	ENSGACG00000008462	*tubg1*	6189885	6195320	tubulin, gamma 1
		2	ENSGACG00000008473	*si:ch211-18i17.2*	6197763	6222667	pleckstrin homology, MyTH4 and FERM domain containing H3
		3	ENSGACG00000008483	*cntnap1*	6237259	6246630	contactin associated protein 1
		4	ENSGACG00000008492	*ezh1*	6251070	6261579	enhancer of zeste 1 polycomb repressive complex 2 subunit
		5	ENSGACG00000008501	*ramp2*	6265046	6267944	receptor (G protein-coupled) activity modifying protein 2
		6	ENSGACG00000008510		6275474	6275799	
		7	ENSGACG00000008514		6277328	6279125	
		8	ENSGACG00000008517	*c1ql3b*	6309449	6320441	complement component 1, q subcomponent-like 3b
		9	ENSGACG00000008519	*ccdc43*	6382036	6384675	coiled-coil domain containing 43

		10	ENSGACG00000008523	*fzd2*	6393118	6394227	frizzled class receptor 2
		11	ENSGACG00000008527	*mylk5*	6409942	6413758	myosin, light chain kinase 5
		12	ENSGACG00000008532	*si:ch73-141c7.1*	6416503	6418554	si:ch73-141c7.1
		13	ENSGACG00000008535	*hsd17b1*	6419439	6421734	hydroxysteroid (17-beta) dehydrogenase 1
		14	ENSGACG00000008544	*zgc:153952*	6439379	6447745	zgc:153952
		15	ENSGACG00000008553	*atp6v0a1a*	6456751	6466815	ATPase, H+ transporting, lysosomal V0 subunit a1a
		16	ENSGACG00000008605	*PTRF*	6468622	6478671	polymerase I and transcript release factor
		17	ENSGACG00000008607	*stat3*	6484785	6492597	signal transducer and activator of transcription 3 (acute-phase response factor)
		18	ENSGACG00000008634	*stat5a*	6519466	6529816	signal transducer and activator of transcription 5a
		19	ENSGACG00000008641	*si:ch211-210g13.5*	6567077	6585055	si:ch211-210g13.5
		20	ENSGACG00000008648	*kcnh4a*	6590904	6602856	potassium voltage-gated channel, subfamily H (eag-related), member 4a

**O**	chrII	1	ENSGACG00000015507	*kif18a*	9100586	9119396	kinesin family member 18A
		2	ENSGACG00000015505		9086923	9092138	
		3	ENSGACG00000015510	*mettl15*	9139122	9161054	methyltransferase like 15
		4	ENSGACG00000015502	*bdnf*	9073816	9074815	ribosomal protein, large P2, like
		5	ENSGACG00000015500	*lin7c*	9068964	9070913	lin-7 homolog C (C. elegans)
		6	ENSGACG00000015499	*rplp2l*	9063720	9064814	ribosomal protein, large P2, like
**P**	chrI	1	ENSGACG00000009072	*grik4*	8598417	8683492	glutamate receptor, ionotropic, kainate 4
**Q**	chrIX	1	ENSGACG00000018024	*ugt8*	12576700	12584061	UDP glycosyltransferase 8
		2	ENSGACG00000018022	*ndst3*	12647423	12676796	N-deacetylase/N-sulfotransferase (heparan glucosaminyl) 3
**R**	chrI	1	ENSGACG00000014605		25581534	25587141	
		2	ENSGACG00000014627	*cbsb*	25574232	25581159	cystathionine-beta-synthase b

		3	ENSGACG00000014641		25563835	25564550	
		4	ENSGACG00000014600	*zgc:172122*	25627729	25630447	
		5	ENSGACG00000014598		25633219	25652062	
**S**	chrXVI	1	ENSGACG00000005749	*WDSUB1*	11001204	11010414	WD repeat, sterile alpha motif and U-box domain containing 1
		2	ENSGACG00000005757	*TANC1*	11011679	11064194	tetratricopeptide repeat, ankyrin repeat and coiled-coil containing 1
		3	ENSGACG00000005734	*BAZ2B*	10980019	10999671	bromodomain adjacent to zinc finger domain 2B

Table S5: Characterization of the 42 core SNPs and frequency of the acidic alleles in marine stickleback (marine samples pooled). The SNPs are sorted by decreasing magnitude of B-A differentiation. The colors coding indicates whether at a given SNP, the major allele in the sea coincides with the one typical of the basic (blue) or acidic (red) populations.

Chr	SNPpos	Mean B-A AFD	Acidic allele (based on global FW pool)	Basic allele (based on global FW pool)	Marine minor allele (based on the 30 marine individuals)	Marine major allele (based on the 30 marine individuals)	Marine minor allele count	Marine major allele count	% Acidic allele in marine pop	% Basic allele in marine pop
chrIX	13360688	0.863	G	C	G	C	3	143	0.021	0.979
chrI	879044	0.826	C	A	A	C	25	77	0.755	0.245
chrIV	26641811	0.818	G	A	G	A	3	171	0.017	0.983
chrVII	13825503	0.800	C	T	C	T	46	102	0.311	0.689
chrXX	10619356	0.793	T	C	T	C	6	117	0.049	0.951

chrV	3953444	0.791	A	G	A	G	31	119	0.207	0.793
chrIV	28685877	0.791	G	A	G	A	5	141	0.034	0.966
chrIV	12031152	0.787	C	G	C	G	8	143	0.947	0.053
chrXIV	7260519	0.784	A	G	A	G	31	110	0.220	0.780
chrXVII	6782419	0.781	C	A	A	C	1	134	0.993	0.007
chrVII	19986534	0.779	G	A	G	A	67	75	0.472	0.528
chrXV	16209497	0.778	T	A	T	A	11	115	0.087	0.913
chrXVII	6492561	0.776	A	G	G	A	29	127	0.814	0.186
chrXI	6536822	0.769	T	G	T	G	2	152	0.013	0.987
chrII	9113855	0.760	G	A	G	A	36	120	0.231	0.769
chrI	8680374	0.757	G	A	G	A	29	146	0.166	0.834
chrIX	12615477	0.754	T	C	C	T	46	112	0.709	0.291

chrI	25584840	0.753	A	T	A	T	10	137	0.068	0.932
chrXVI	11017185	0.752	C	T	C	T	27	83	0.245	0.755
chrX	7862245	0.747	A	G	A	G	31	118	0.208	0.792
chrVII	3036974	0.741	C	A	C	A	65	70	0.481	0.519
chrVII	13908696	0.738	C	G	C	G	43	102	0.297	0.703
chrIV	7849606	0.732	T	G	T	G	23	106	0.178	0.822
chrI	21308259	0.729	C	A	C	A	46	67	0.407	0.593
chrXV	881351	0.728	A	G	A	G	5	136	0.035	0.965
chrIV	8523376	0.727	A	C	A	C	40	87	0.315	0.685
chrVII	14277048	0.725	C	A	C	A	11	157	0.065	0.935
chrIX	13102705	0.720	C	A	A	C	1	189	0.995	0.005
chrIV	7010171	0.718	C	T	C	T	70	80	0.467	0.533

chrVII	5378300	0.718	A	G	A	G	18	40	0.310	0.690
chrX	11336552	0.717	A	G	G	A	6	124	0.954	0.046
chrIII	5077315	0.716	T	G	T	G	25	61	0.291	0.709
chrVII	22905391	0.713	A	C	C	A	62	82	0.569	0.431
chrX	9814424	0.712	A	G	G	A	1	108	0.991	0.009
chrXVII	1404567	0.712	T	A	T	A	10	90	0.100	0.900
chrXX	13066370	0.709	C	A	C	A	48	110	0.304	0.696
chrXX	9770450	0.709	C	T	C	G	5	112	0.045	0.000
chrVII	12965594	0.706	C	T	C	T	29	45	0.392	0.608
chrXX	9376877	0.704	T	G	T	G	47	90	0.343	0.657
chrV	8832730	0.700	G	A	G	A	45	95	0.321	0.679
chrIV	10565725	0.700	A	C	A	C	33	125	0.209	0.791

chrXVI	9076916	0.700	A	G	A	G	50	96	0.342	0.658

Figures

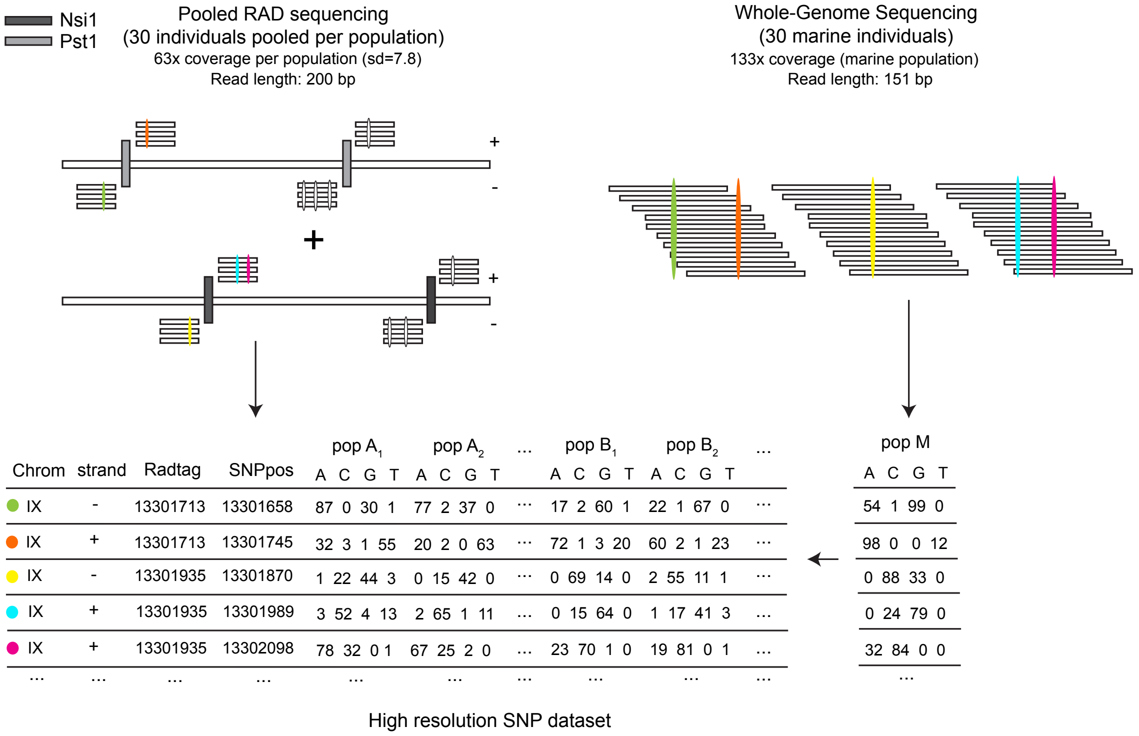

Figure S1: Schematic description of the SNP generation protocol based on pooled RAD and whole-genome sequencing. For the basic and acidic populations, we modified the classical RAD protocol (Baird et al 2008) by performing a parallel digestion of the basic and acidic samples (~ 30 individuals pooled per population) by two restriction enzymes (Nsi1 and Pst1) (top left). For the 30 total marine individuals, we performed whole-genome sequencing (top right). SNPs are visualized as colored ovals. After appropriate filtering steps, a high-resolution SNP dataset was then generated by performing allele counts for each population at each base position (bottom left). Marine allele counts were performed only at the SNPs ascertained in the freshwater samples and added to the SNP matrix (bottom right).

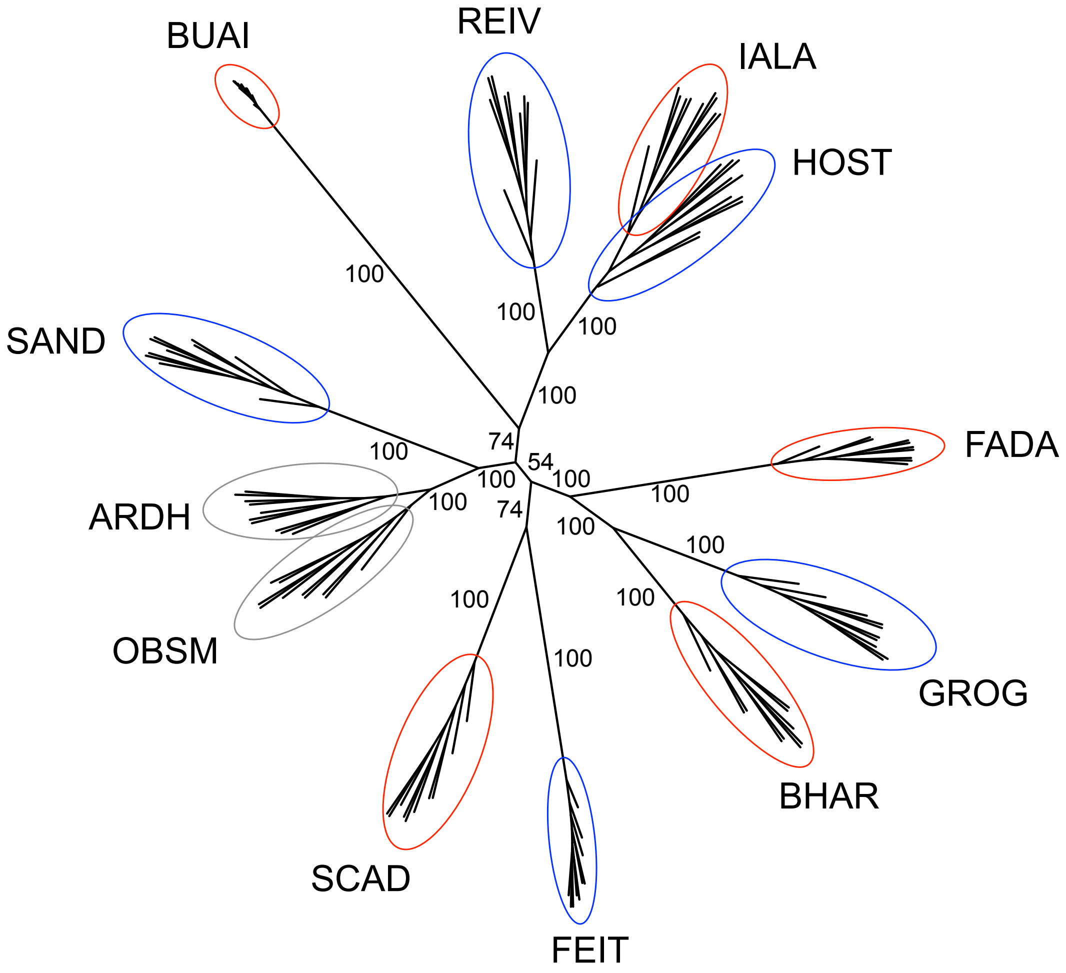

Figure S2: Unrooted nuclear phylogeny based on 15,058 SNPs, using the full ten synthetic individuals generated for each population (instead of a single one, as in Fig. 2A). Color coding is by habitat, as in Fig. 1.

Figure S3

A

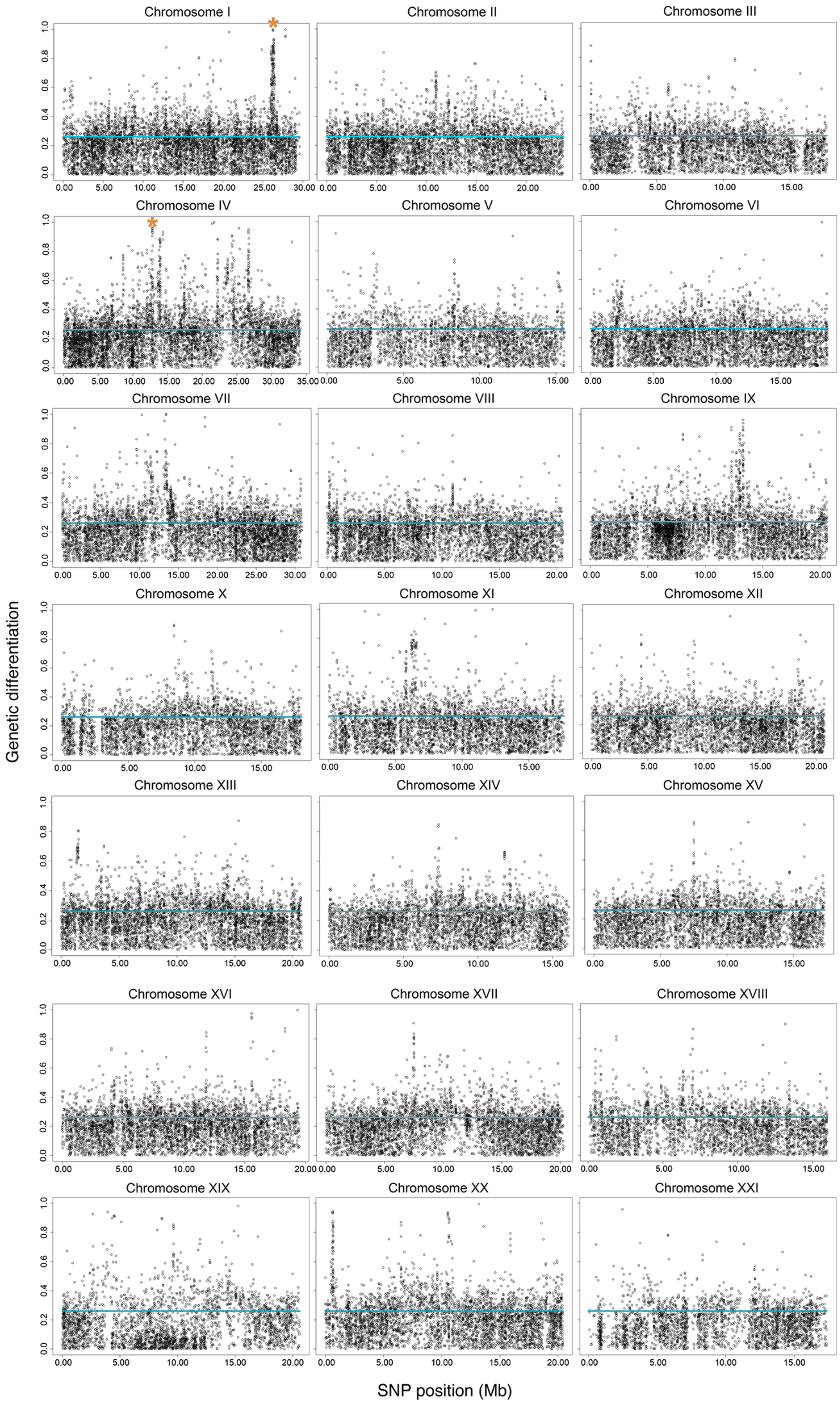

Chromosome I
Chromosome II
Chromosome III
Chromosome IV
Chromosome V
Chromosome VI
Chromosome VII
Chromosome VIII
Chromosome IX
Chromosome X
Chromosome XI
Chromosome XII
Chromosome XIII
Chromosome XIV
Chromosome XV
Chromosome XVI
Chromosome XVII
Chromosome XVIII
Chromosome XIX
Chromosome XX
Chromosome XXI
Genetic differentiation
SNP position (Mb)

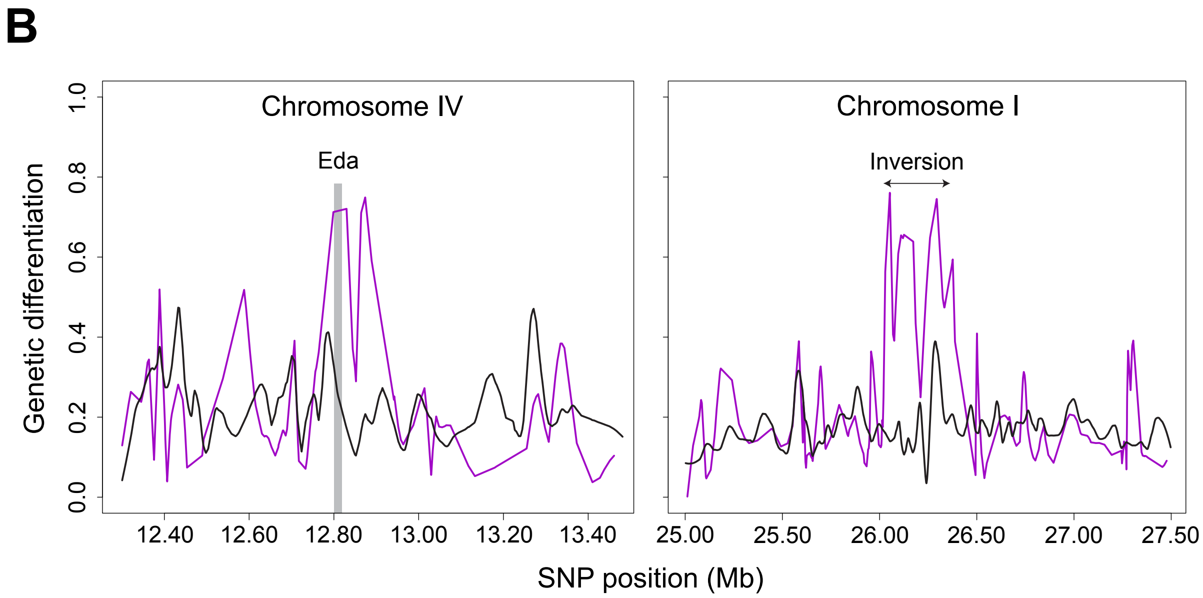

Figure S3: (A) Profiles of marine-freshwater genetic differentiation, quantified as absolute allele frequency difference, across the 21 treespine stickleback chromosomes. The blue line indicates the median (0.26) across all genome-wide SNPs. For this analysis, all basic and acidic populations were combined to a global freshwater pool and compared to the marine population (i.e., the OBSM and ARDH samples pooled). SNP detection followed a strategy differing from the one underlying the SNP data set used for the main investigation of basic-acidic differentiation: at each base position covered by the RAD tags of the freshwater fish, we considered the full marine nucleotide coverage (average: 133x), and a nucleotide sample of exactly the same size drawn at random from the freshwater pool. A variable position then qualified as SNP when these two samples combined exhibited a MAF of at least 0.05, and when read coverage was within 50-240 for the marine pool and within 200-2800 for the full freshwater pool. For SNPs satisfying these criteria, we then performed base counts for each population (1 marine, 10 freshwater). These data were saved in a SNP matrix (available on Dryad) and used to calculate overall marine versus freshwater allele frequency differences at all SNPs. Dark orange

asterisks indicate the position of the chromosome I inversion and the Eda locus (chromosome IV), regions well-known to be under divergent selection between marine and freshwater stickleback (e.g., Hohenlohe et al. 2010; Jones et al. 2012; Roesti et al. 2014; Terekhanova et al. 2014; Nelson & Cresko 2018). (B) Patterns of genetic differentiation around the same two classical loci of marine-freshwater differentiation, based on the SNPs ascertained using the freshwater populations only (i.e., as in our main analyses). The purple and black lines here represent marine-freshwater and basic-acidic (B-A) differentiation (mean AFD across all corresponding population comparisons). Note that B-A differentiation is low at both loci, consistent with the sharing of haplotypes *universally* favorable in freshwater (that is, favorable in both basic and acidic lakes). The profiles are smoothed to reduce complexity; the magnitude of marine-freshwater differentiation at individual SNPs is even higher.

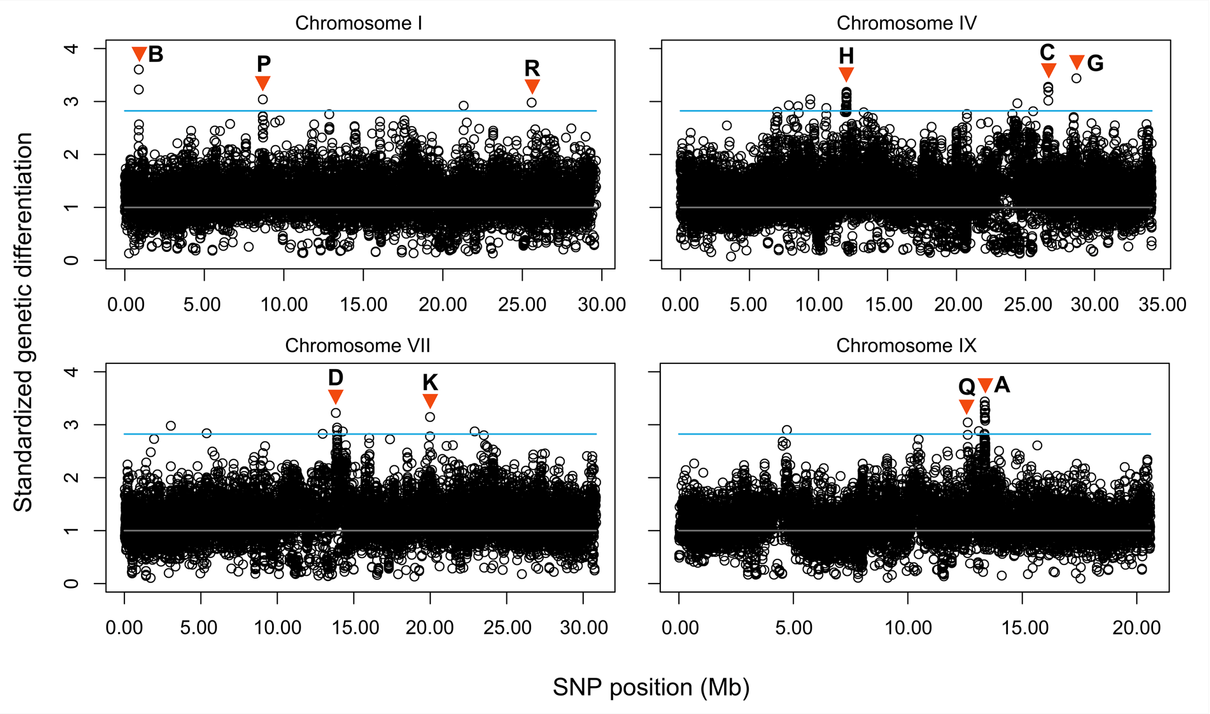

Figure S4: Check of the robustness of identifying genomic regions of highly parallel basic-acidic differentiation (top core SNPs) by integrating AFD data from multiple population comparisons without taking differences among comparisons in their overall level of differentiation into account (the outcome of this type of data integration chosen for our study is hereafter called 'AFD_{RAW}'). For this, we repeated the integration of AFD data across the B-A comparisons by first standardizing all AFD values from a given population comparison by the genome-wide median AFD value for that comparison (yielding 'AFD_{STAND}'). As a critical check for the consistency between the non-standardized and standardized identification of the core SNPs, we then retrieved the top 42 SNPs based on AFD_{STAND} (corresponding to a threshold of 2.8241, indicated by the blue line above) and determined the degree of overlap with the 42 SNPs identified based on AFD_{RAW} (i.e., the normal core SNPs in the main paper). The congruence between these two approaches was very high: 36 (86%) of

the 42 top SNPs identified using the AFD_{STAND} approach proved identical at the precise base pair level with our core SNPs. The similarity between the two approaches is visualized above for the four chromosomes harboring the highest number of top core SNPs. Here the dots represent the average B-A differentiation across the multiple comparisons at each SNP, as obtained after standardizing each comparison by its genome-wide median (hence the Y-axis scale no longer ranges from zero to one, contrary to AFD_{RAW} in Fig. 3A). Dark orange triangles indicate the position of the core SNPs on these chromosomes, as based on the AFD_{RAW} approach. These SNPs also emerge as the regions of strongest differentiation on each chromosome when using the AFD_{STAND} method. The high consistency between the two approaches to integrating differentiation data from multiple population comparisons justifies using the mathematically simpler one (i.e., no standardization). A further reason why we base our identification of top core SNPs on AFD_{RAW} is that the core SNPs of these genome regions proved completely fixed for alternative alleles (i.e., AFD = 1) in some basic-acidic population comparisons. Since AFD cannot increase beyond one even when the overall level of differentiation continues to increase, standardization by the latter may lead to the underestimation of genetic differentiation. Given that the overall level of differentiation was reasonable similar among all B-A comparisons anyway (Table S2), the non-standardized approach appeared superior to us.

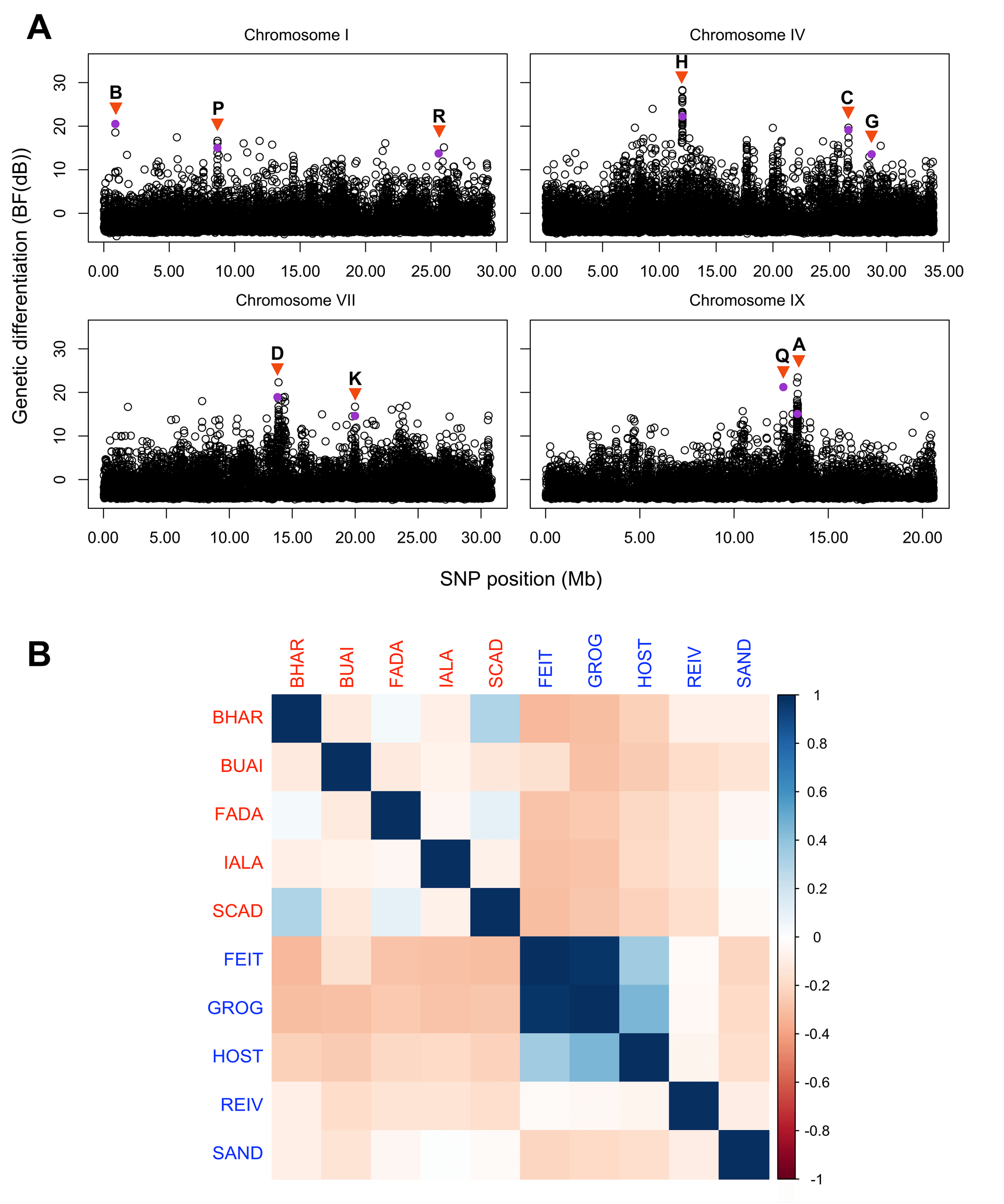

Figure S5: BayPass analysis. (A) Check of the robustness of identifying genomic regions of highly parallel basic-acidic differentiation (i.e., core SNP regions) by integrating AFD data from multiple population comparisons (our method presented in the paper) against an ecotype-related outlier SNP scan using BayPass (Gautier

2015). For the BayPass analysis, we used the same SNP data set as for our method, a binary scoring for the ecotype covariate (1 = basic, 2 = acidic), and the same parameters as described in Leblois et al. 2017. As a critical check for the consistency between the two approaches, we focused on the variable BF(dB) from the BayPass output expressing for each SNP the strength of association to basic versus acidic ecotype. Based on this variable, we retrieved the top 1% of the SNPs and determined visually what proportion of our 42 core SNP regions coincided with regions containing one or multiple of these BayPass ‘outliers’. This check revealed a high congruence between the methods: 36 (86%) out of our 42 total core SNP regions also emerged unambiguously as BayPass outlier regions. In (A), this congruence is visualized for the same four chromosomes as in Fig. S4. Here, dark orange triangles indicate a subsample of our 19 top core SNP regions (i.e., core SNP showing AFD > 0.75 in the combined B-A comparison), with the precise core SNPs shown as purple dots. The consistency between the methods clearly confirms the robustness of our method. (B) Correlation matrix based on scaled population allele frequencies covariances estimated by BayPass. The color shade expresses the magnitude of positive or negative correlation for a given population pair. This matrix generally reveals weak among-population correlations in allele frequencies, as expected from the independent evolution of the lake populations indicated by our other analyses (phylogenies, ordination). A potential lack of independence is suggested only for the FEIT and GROG basic population pair. This appears plausible, given that the outlet of FEIT could not be determined with confidence (Fig. 1A).

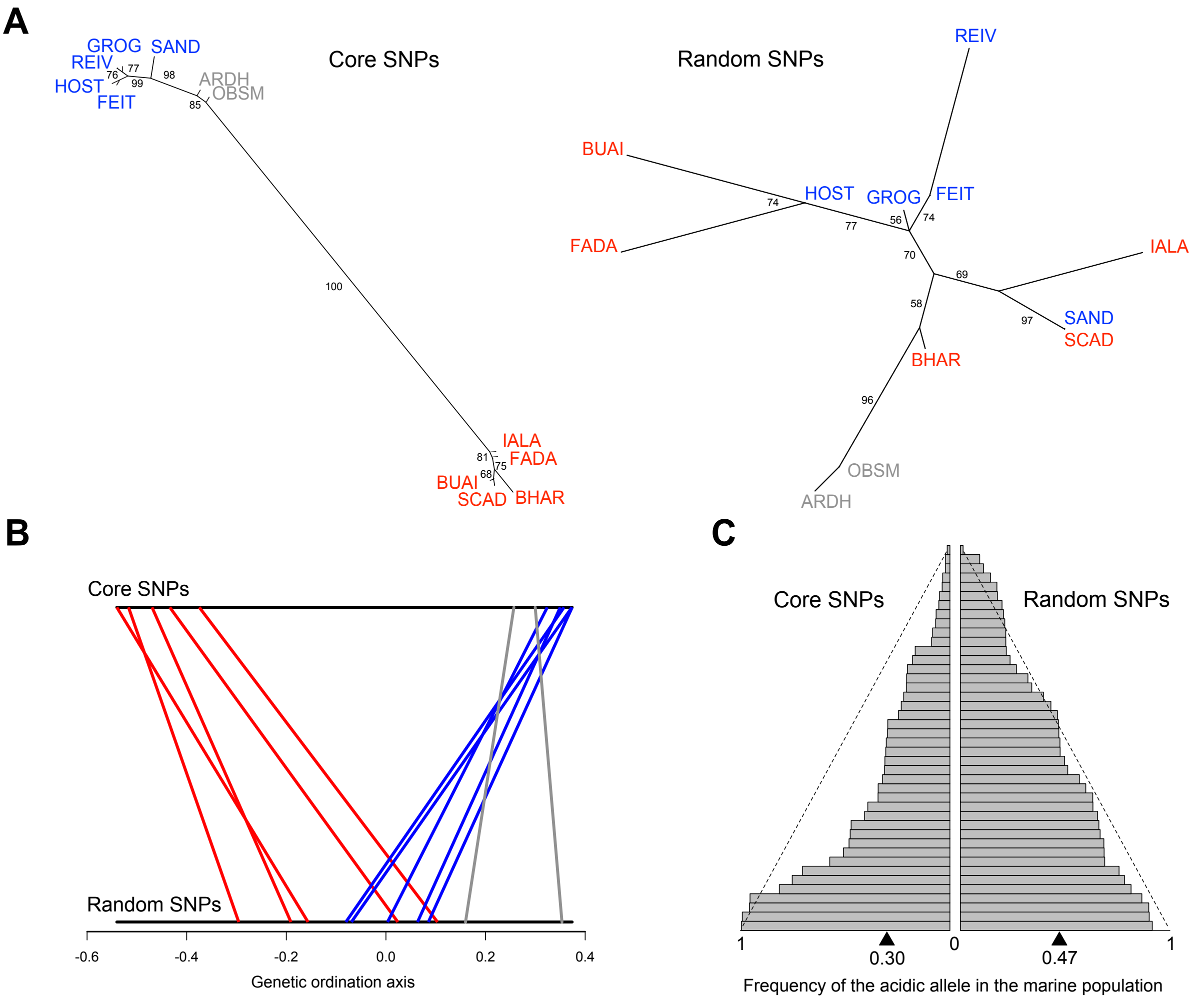

Figure S6: Replication of the analyses presented in Fig. 4 based on a new set of random SNPs. These were chosen at random among all the SNPs displaying an AFD inferior to 0.5 in the integrated B-A comparison. We here thus controlled much less effectively for the selective neutrality of the random SNPs (recall that the random SNPs used in the paper were required to fall within a very narrow AFD window around the genome-wide median). Apart from the different set of random SNPs, all analytical conventions and graphing styles correspond to those underlying Fig. 4. Note that using a different set of random SNPs leads to similar results supporting the same conclusion, even when enforcing the selective neutrality of these SNPs less strictly.

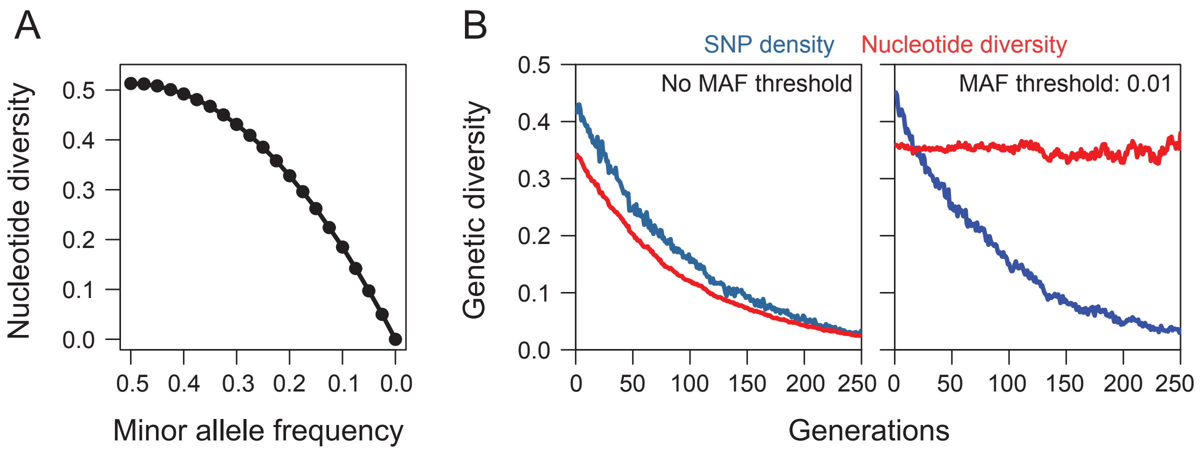

Figure S7: Exploration of the sensitivity and robustness of SNP density, the metric of within-population genetic diversity employed in our study, in comparison to nucleotide diversity (π; Nei & Li 1979). (A) Shows nucleotide diversity, computed as the fraction of nucleotide mismatches among all possible pairwise nucleotide permutations, along the continuum of decreasing genetic variation as defined by the frequency of the minor allele (MAF) among 40 total nucleotides. The left end of the X-axis represents two alleles in perfectly balanced proportion (20 vs. 20), while the right end corresponds to the fixation for one allele (40 vs. 0). This numerical analysis reveals a non-linear response of nucleotide diversity to the loss of genetic variation at a polymorphism: a given allele frequency reduction causes a relatively weak change in nucleotide diversity in the MAF range representing alleles in relatively balanced proportion, whereas an allele frequency reduction of the same magnitude drives a strong change in nucleotide diversity in the MAF range in which one allele is rare. In (B), SNP density – a genetic diversity metric derived directly from the MAF, and nucleotide diversity were applied to a simulated population to examine how these metrics respond to a reduction in genetic diversity across numerous loci. We here simulated a population of 100 haploid individuals and 1000 unlinked bi-allelic SNPs. At each SNP, genotypes were initially drawn at random from a uniform distribution

(for an empirical justification see Fig. 4C). The population then experienced a loss of diversity over 250 generations by drift, achieved by re-sampling each SNP with replacement to the original population size. In each generation, a subsample of 40 nucleotides (similar to the minimum coverage threshold used in our empirical analyses) was drawn at each SNP. Based on these subsamples, nucleotide diversity was calculated as described above and averaged over all SNPs. SNP density was calculated as the proportion of SNPs for which the subsample satisfied a MAF threshold of 0.3 (the same threshold as in our empirical analyses of genetic diversity). This algorithm was carried out in two modes: either by accepting *all* SNPs for genetic diversity calculation (visualized in the left panel), or by first filtering the subsample at each SNP according to a mild MAF threshold of 0.01, and calculating the two diversity metrics only based on those SNPs satisfying this threshold (shown in the right panel). With a sample size of 40 nucleotides, this latter MAF threshold eliminated all monomorphic SNPs plus the singletons.

The left panel of (B), involving no low-MAF filter, shows that as diversity declines (i.e., the SNPs move stochastically toward monomorphism), SNP density and nucleotide diversity are tightly correlated. Consistent with the reduced sensitivity of nucleotide diversity to allele frequency shifts in the high-MAF range identified in (A), however, the decline in nucleotide diversity is slightly less steep than the decline in SNP density. At least for SNPs showing allele frequencies broadly consistent with a uniform distribution, SNP density thus captures the loss of diversity more sensitively than nucleotide diversity.

The right panel of (B) further reveals a dramatic influence of low-MAF filtering on nucleotide diversity but not SNP density: excluding monomorphic SNPs and

singletons renders nucleotide diversity almost completely insensitive to diversity reduction. Although the mild MAF filter (0.05) applied to the global pool of all our freshwater populations to exclude sequencing error is unlikely to eliminate low-diversity sites within the populations as radically as the low-MAF filter in this second simulation mode, this simulation nevertheless makes clear that MAF thresholds can affect the estimation of genetic diversity by nucleotide diversity substantially. The reason is that such thresholds alter both total SNP number and the relative fraction of those SNPs for which nucleotide diversity exhibits the highest sensitivity (i.e., the low-MAF range, see A). By contrast, SNP density is not materially influenced by MAF filtering.

Overall, we conclude that SNP density, the metric of genetic diversity adopted in our work, not only captures diversity loss more sensitively than nucleotide diversity, it also represents a diversity metric highly robust to MAF filtering. Clearly, the use of SNP density in our analytical context is well motivated.

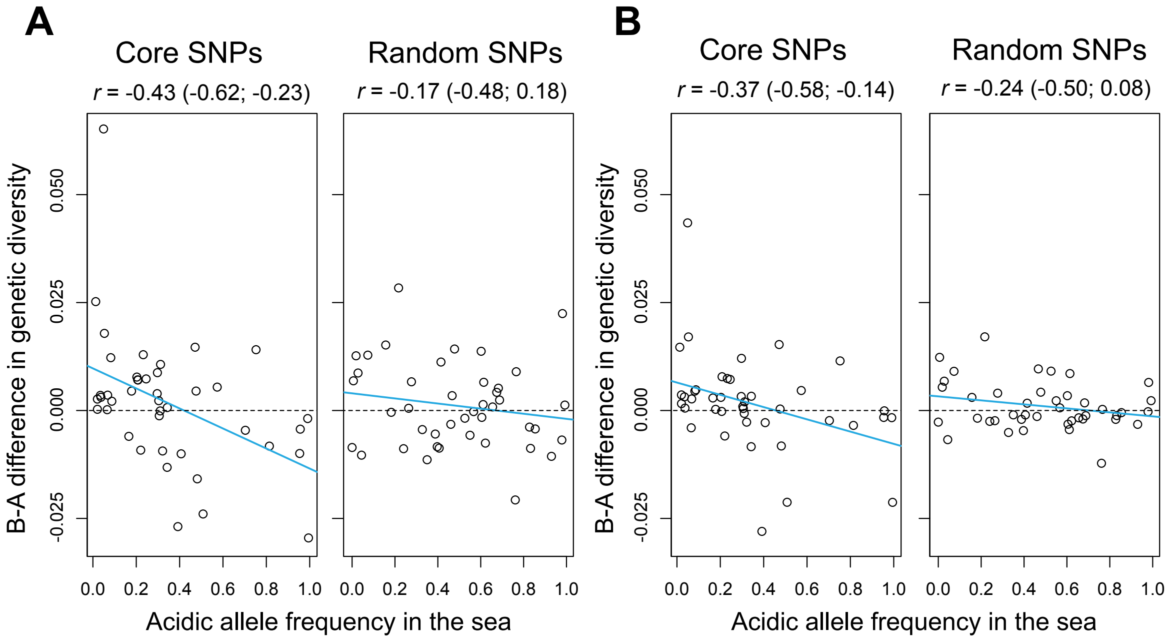

Figure S8: Robustness of the selective sweep analysis. In (A), this analysis was repeated by considering SNP density across a narrower chromosome window (20 kb as opposed to 40 kb) around the focal SNPs (core and random). In (B), we performed the selective sweep analysis by applying a different MAF threshold for determining the number of high-MAF SNPs (0.2 as opposed to 0.3). Further MAF thresholds examined included 0.15 and 0.25, producing similar results, although for theoretical reasons mentioned in the paper, high MAF thresholds should reveal selective sweeps most reliably. All other analytical conventions and graphing styles follow those underlying Fig. 6. Collectively, these supplementary analyses confirm a strong relationship between genetic diversity and allele frequencies in the sea for the core SNPs only, consistent with our conclusion of selective sweeps drawn in the paper.

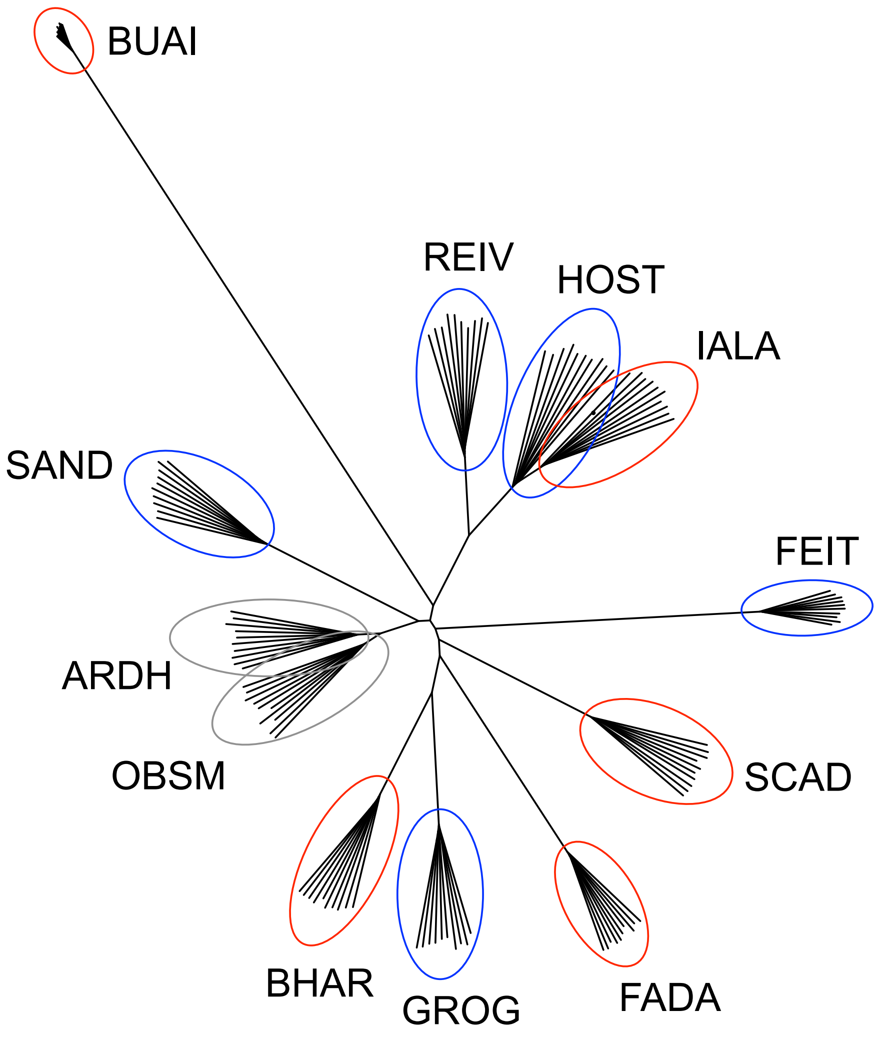

Figure S9: Unrooted neighbor-joining nuclear phylogeny based on 15,058 SNPs, using the full ten synthetic individuals generated for each population. Color coding is by habitat, as in Fig. 1.

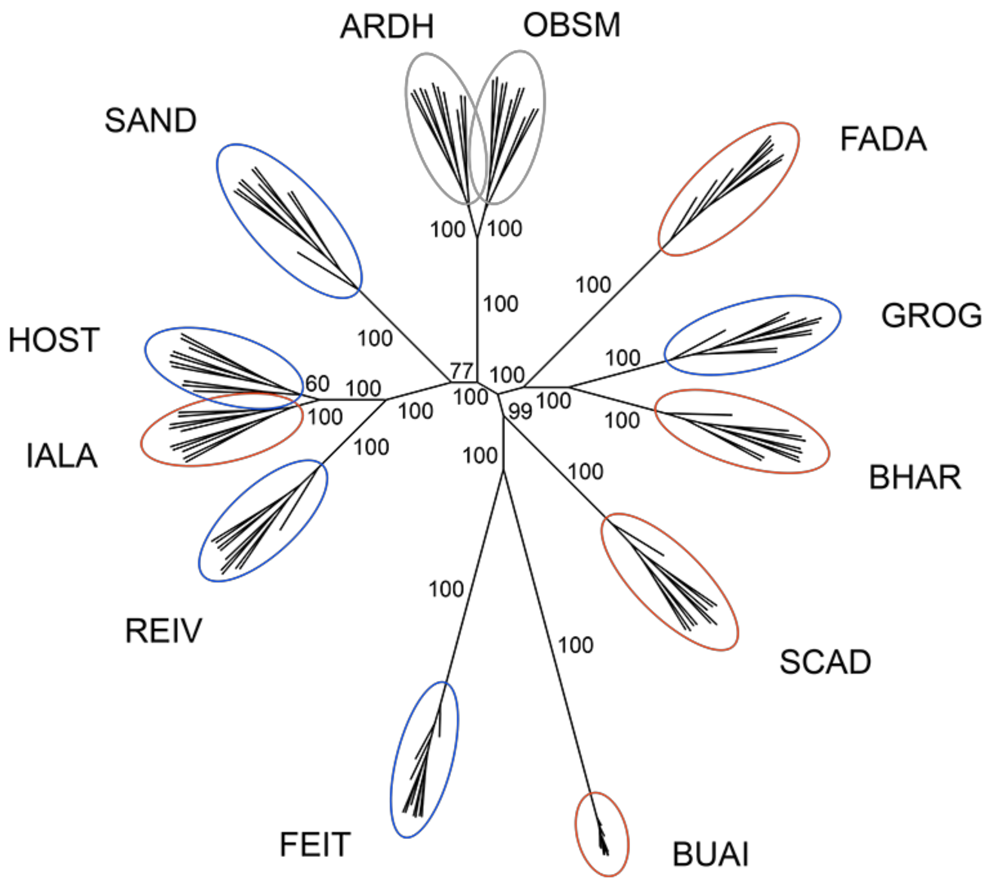

Figure S10: Unrooted nuclear phylogeny based on 68,245 SNPs, using the full ten synthetic individuals generated for each population. Color coding is by habitat, as in Fig. 1.

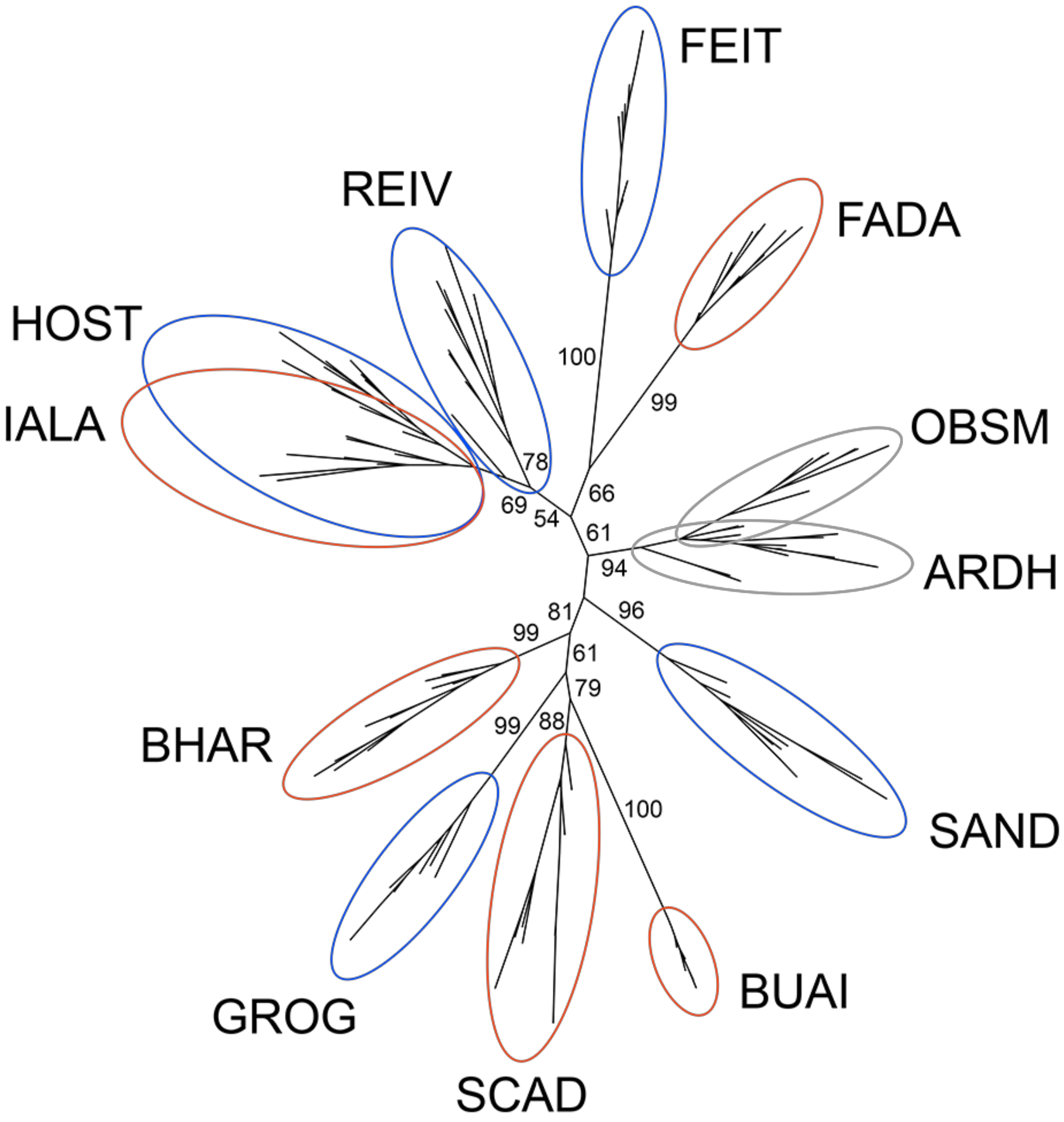

Figure S11: Unrooted nuclear phylogeny based on just 227 SNPs spaced by at least 1 Mb, with ten synthetic individuals generated for each population. Color coding is by habitat, as in Fig. 1. Note that despite this low number of markers, the populations are generally still monophyletic, and the position of basic and acidic populations across the tree remains random, consistent with the independent evolution of the freshwater populations.

Figure S12

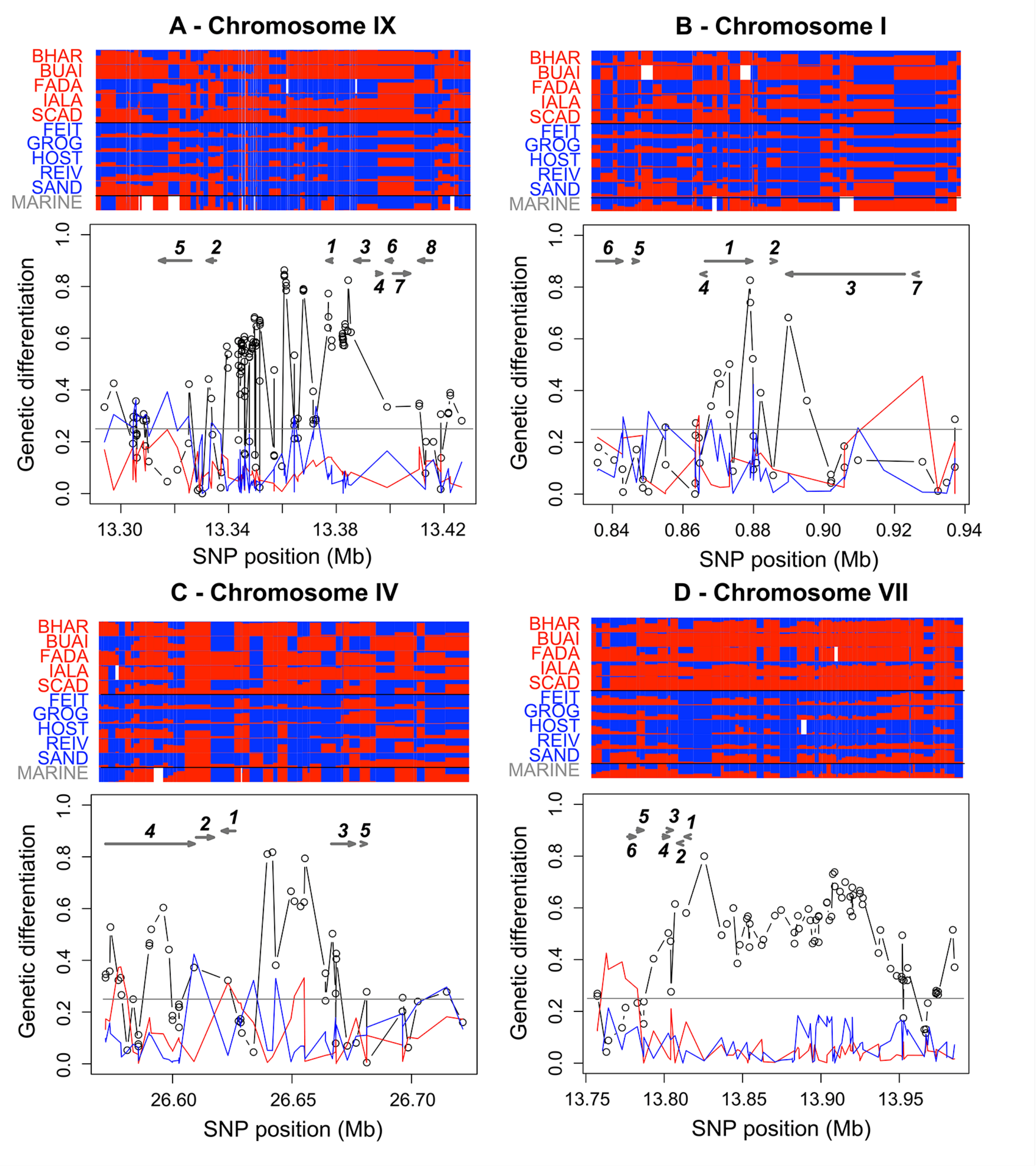
A - Chromosome IX
B - Chromosome I
C - Chromosome IV
D - Chromosome VII
BHAR
BUAI
FADA
IALA
SCAD
FEIT
GROG
HOST
REIV
SAND
MARINE
Genetic differentiation
SNP position (Mb)
13.30
13.34
13.38
13.42
0.84
0.86
0.88
0.90
0.92
0.94
26.60
26.65
26.70
13.75
13.80
13.85
13.90
13.95

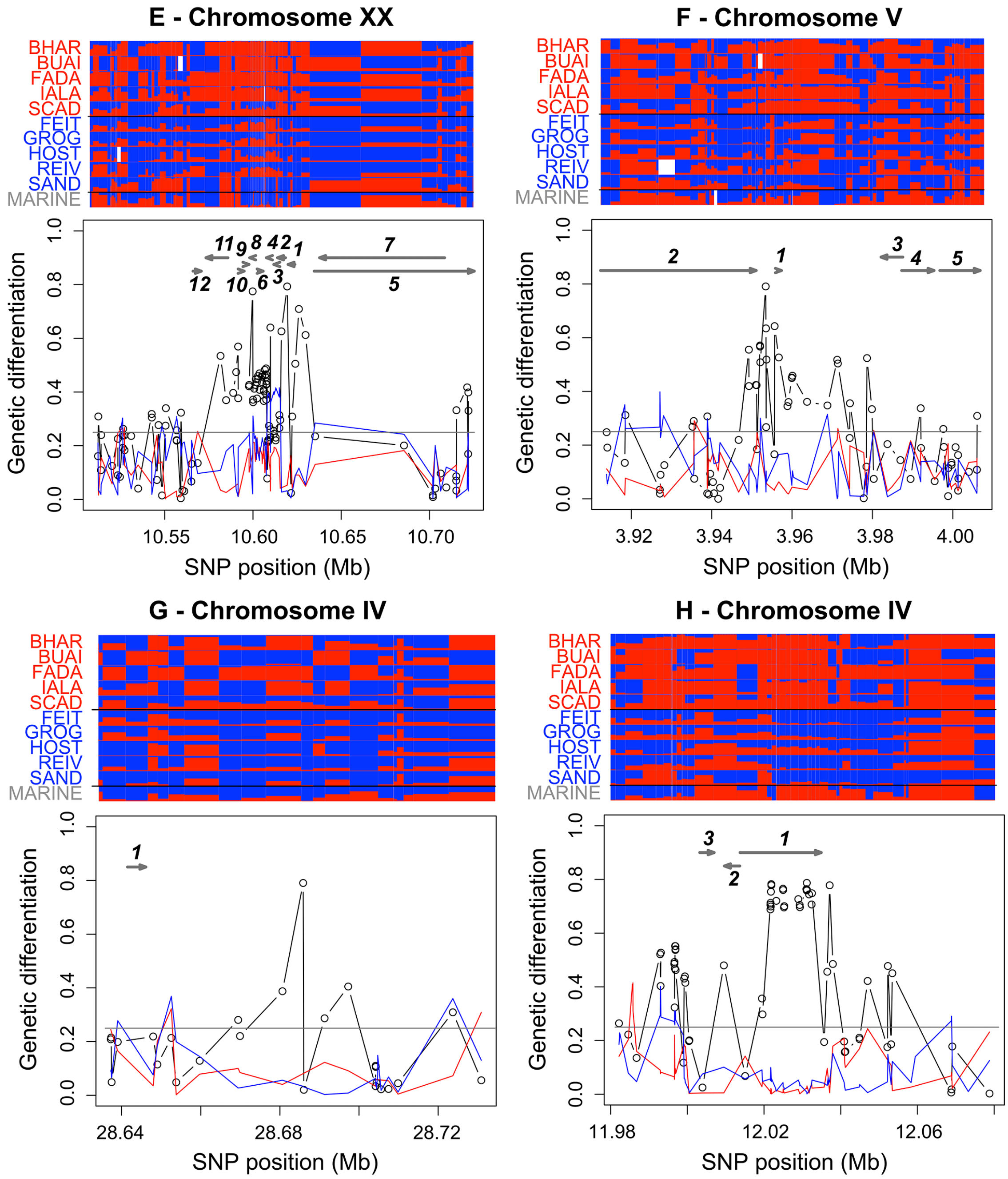
E - Chromosome XX
F - Chromosome V
G - Chromosome IV
H - Chromosome IV
BHAR
BUAI
FADA
IALA
SCAD
FEIT
GROG
HOST
REIV
SAND
MARINE
Genetic differentiation
SNP position (Mb)
10.55
10.60
10.65
10.70
3.92
3.94
3.96
3.98
4.00
28.64
28.68
28.72
11.98
12.02
12.06

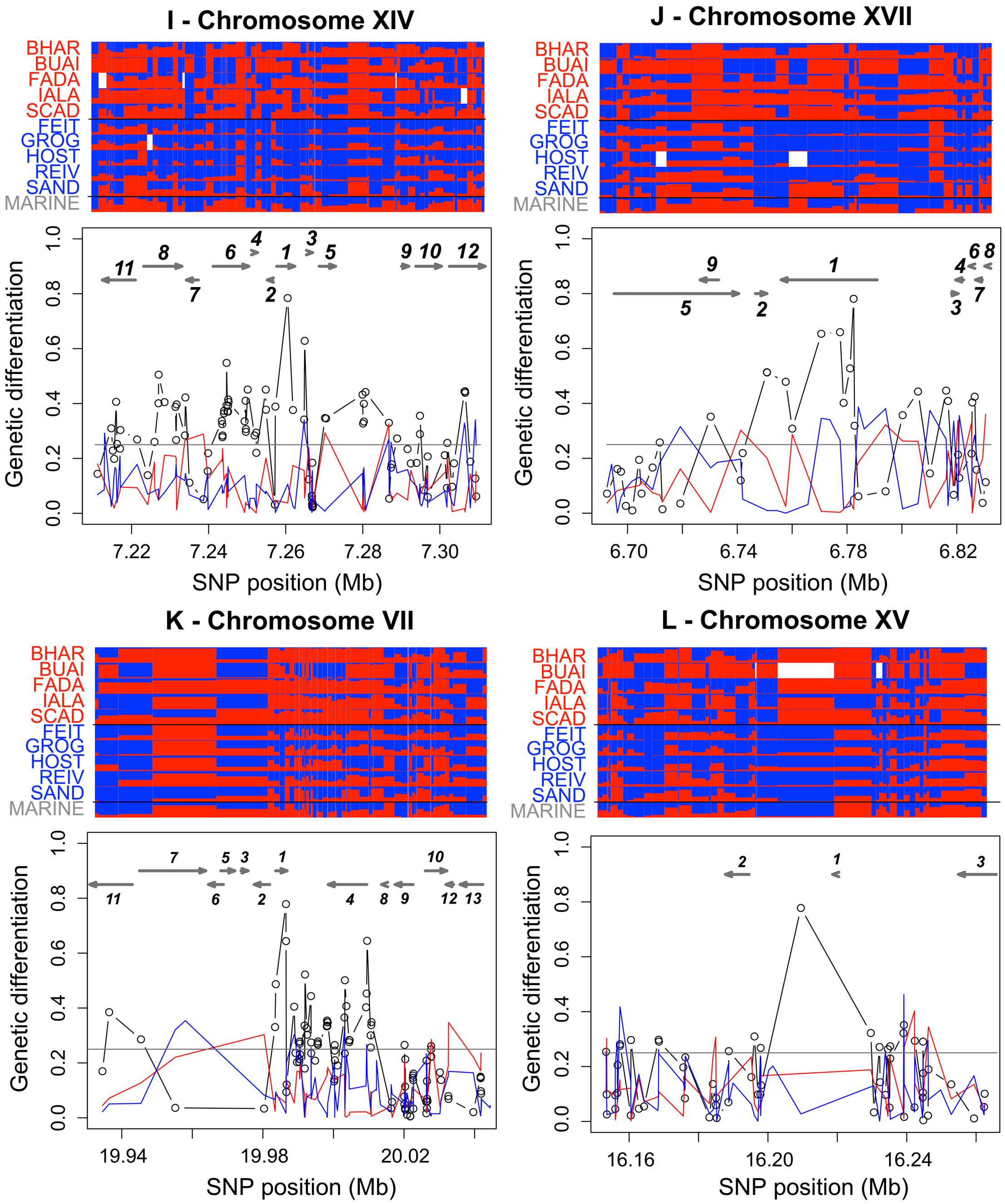

I - Chromosome XIV
J - Chromosome XVII
K - Chromosome VII
L - Chromosome XV
BHAR
BUAI
FADA
IALA
SCAD
FEIT
GROG
HOST
REIV
SAND
MARINE
Genetic differentiation
SNP position (Mb)

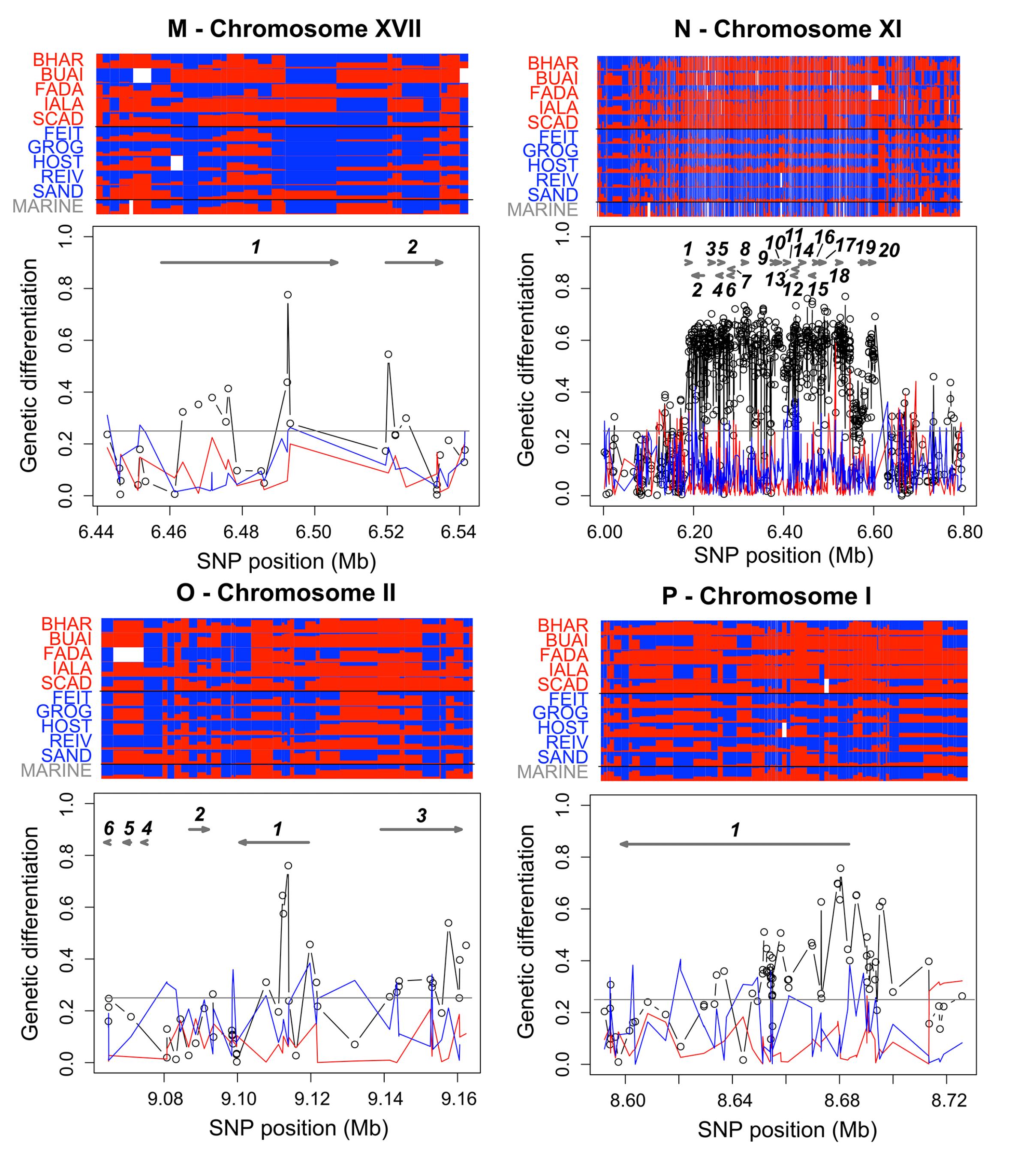
M - Chromosome XVII
N - Chromosome XI
O - Chromosome II
P - Chromosome I
BHAR
BUAI
FADA
IALA
SCAD
FEIT
GROG
HOST
REIV
SAND
MARINE
Genetic differentiation
SNP position (Mb)

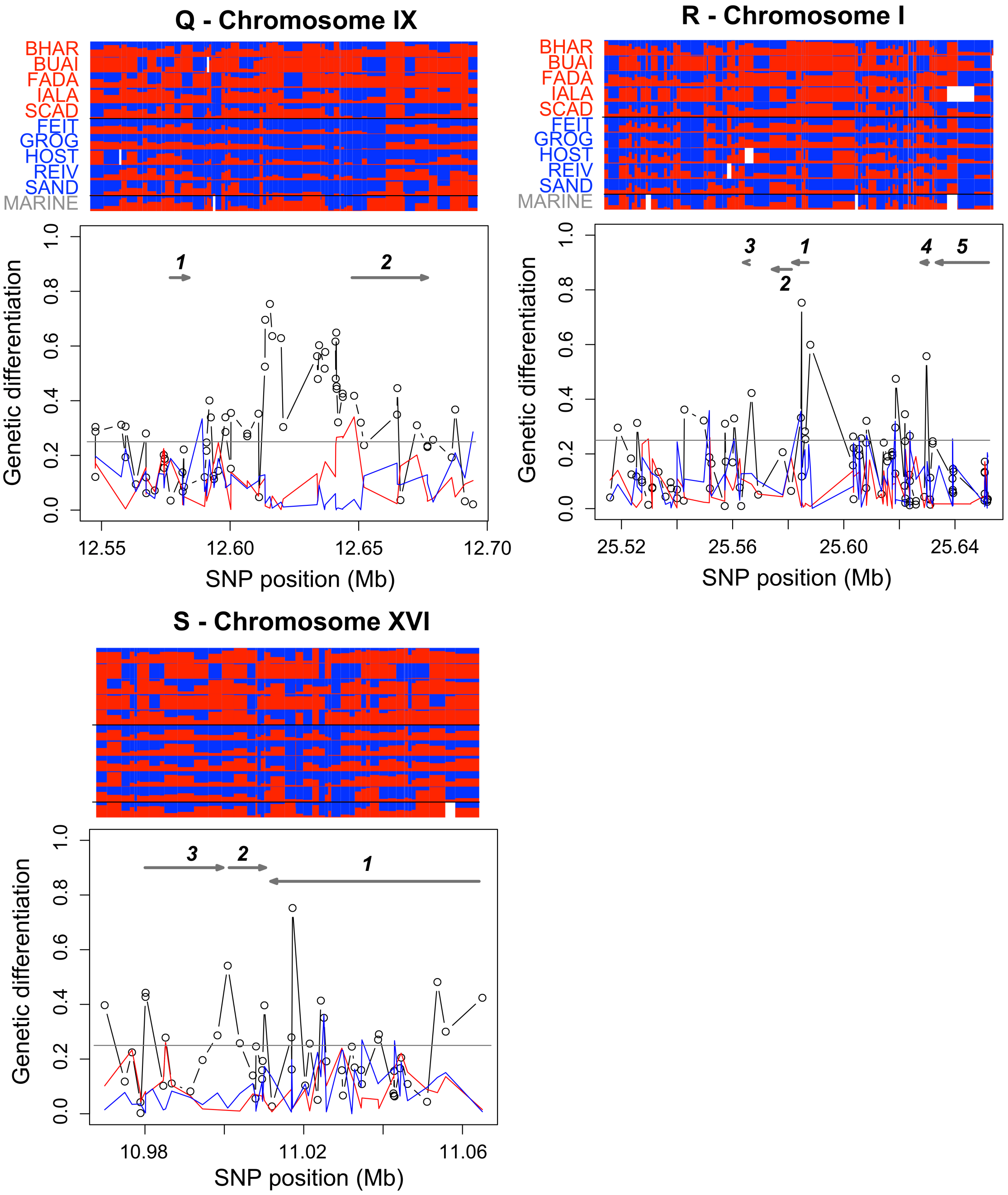

Figure S12: Description of the 19 top core SNPs (mean AFD > 0.75 across the integrated B-A comparison) representing the genomic regions showing the strongest and most consistent basic-acidic differentiation. Regions are ordered by decreasing AFD at the core SNP and are labeled from A to S, consistent with the labeling used

in Table S4. Presentation style follows Fig. 3A, except that genes are numbered to link them to their characterization provided in Table S4.

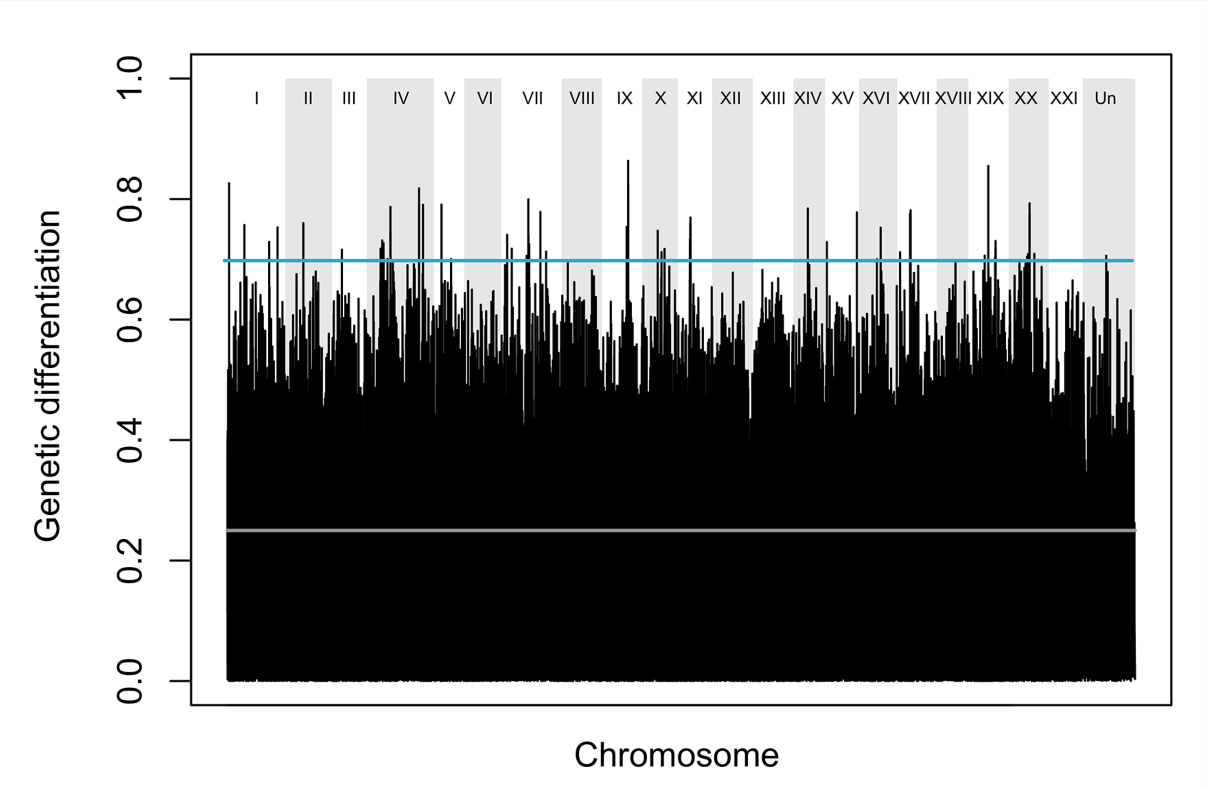

Figure S13: Genome-wide basic-acidic differentiation, as obtained by integrating SNP-specific AFD values across all B-A comparisons. The gray line represents genome-wide median differentiation (0.25), the blue line represents the threshold (0.70) used to identify the core SNPs considered genomic regions of strong and consistent B-A differentiation. Gray and white backgrounds separate the chromosomes. The chromosome 'Un' represents a concatenation of scaffolds not physically anchored to the other chromosomes. The region of high differentiation on chromosome XIX was not considered for further analysis, as this chromosome is the sex chromosome in threespine stickleback.

Figure S14

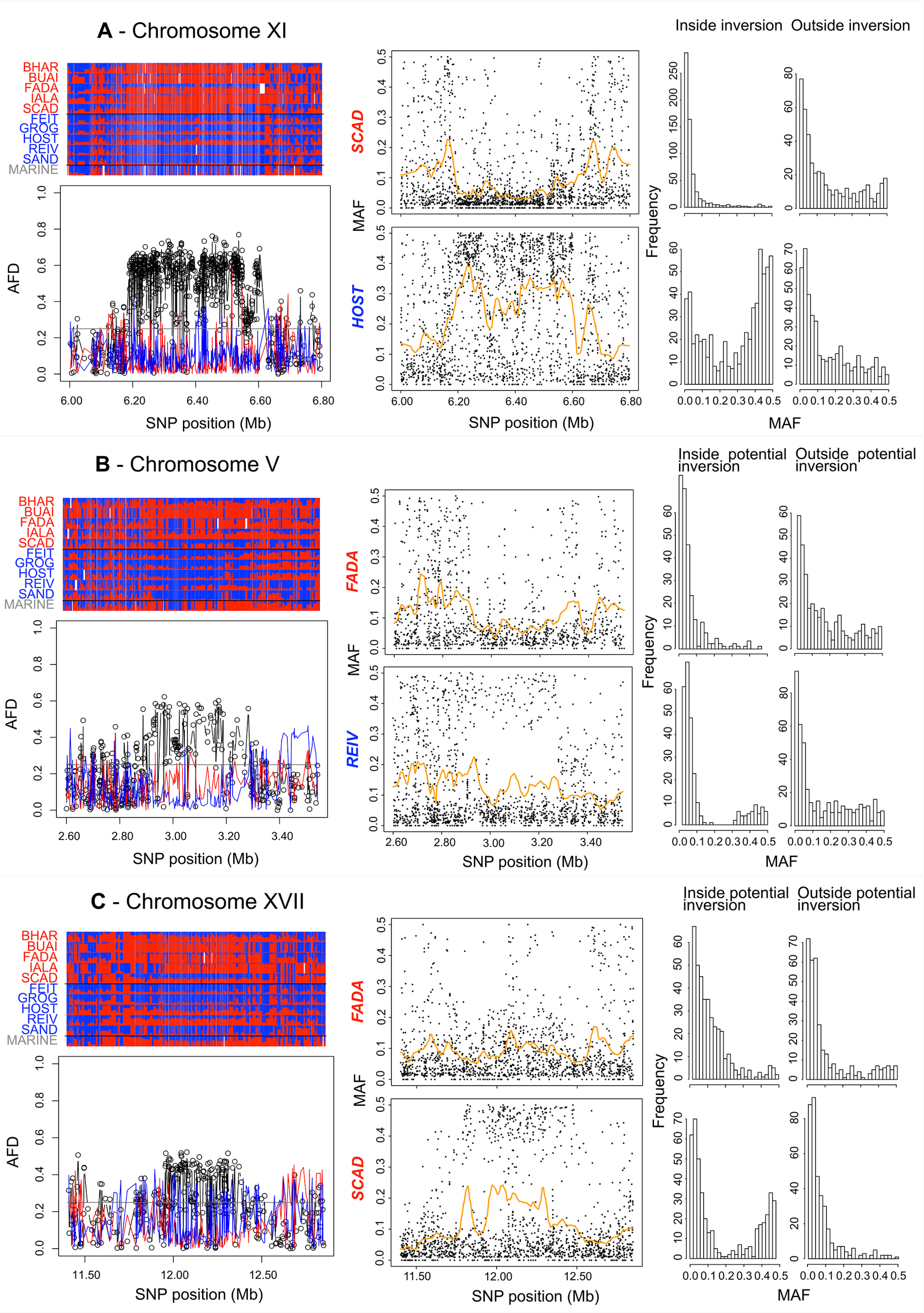
A - Chromosome XI
B - Chromosome V
C - Chromosome XVII
BHAR
BUAI
FADA
IALA
SCAD
FEIT
GROG
HOST
REIV
SAND
MARINE
AFD
MAF
SCAD
HOST
FADA
REIV
SNP position (Mb)
Inside inversion
Outside inversion
Inside potential inversion
Outside potential inversion
Frequency
MAF

Figure S14: Chromosomal inversions produce characteristic patterns in population differentiation and in the frequency of the minor allele, illustrated above for the known inversion on chromosome XI (A), and for two novel potential inversions on the chromosomes V and XVII (B and C). For each (potential) inversion, the bottom panels in the left column present mean genetic differentiation (AFD) profiles for the integrated basic-acidic (black), basic-basic (blue) and acidic-acidic (red) population comparisons, as in Fig. 3A. The dots represent individual SNPs, and the horizontal gray lines indicate genome-wide median differentiation for the integrated B-A comparisons. The top left panels visualize the relative frequencies of the SNPs alleles in each population at all SNPs underlying the differentiation profiles, again following Fig. 3A. The middle column presents the minor allele frequency (MAF) at each SNP position across a chromosome segment around the (potential) inversion. The top panels show MAF for a population (nearly) monomorphic for one specific inversion type, whereas the bottom panels show MAF for a population in which both inversion types occur at relatively balanced frequencies. The right column summarizes the frequency distribution of the MAF across the chromosome segments visualized in the middle column, separately so for the SNPs located inside (left) and outside (right) of the (candidate) inversions (assumed boundaries, from top to bottom: 6.2-6.6 Mb; 2.9-3.3 Mb; 11.9-12.4 Mb). Note that the presence of both (potential) inversion types at a balanced frequency generates a bimodal MAF distribution.

References

Bell, M. F., S. (1994). *The evolutionary biology of the threespine stickleback*, Oxford University Press.

Bierne, N., Gagnaire, P. A. and David, P. (2013). The geography of introgression in a patchy environment and the thorn in the side of ecological speciation. *Curr Zool* 59: 72-86.

Catchen, J., Bassham, S., Wilson, T., Currey, M., O'Brien, C., Yeates, Q. *et al.* (2013). The population structure and recent colonization history of Oregon threespine stickleback determined using restriction-site associated DNA-sequencing. *Mol Ecol* 22: 2864-2883.

Gautier, M. (2015). Genome-wide scans for adaptive differentiation and association analysis with population-specific covariables. *Genetics* 201: 1555-1579

Giles, N. (1983). The possible role of environmental calcium levels during the evolution of phenotypic diversity in Outer Hebridean populations of the three-spined stickleback, *Gasterosteus aculeatus*. *J Zool* 199: 535-544.

Hohenlohe, P. A., Bassham, S., Etter, P. D., Stiffler, N., Johnson, E. A. and Cresko, W. A. (2010). Population genomics of parallel adaptation in threespine stickleback using sequenced RAD tags. *PLoS Genet* 6: e1000862.

Jones, F. C., Chan, Y. F., Schmutz, J., Grimwood, J., Brady, S. D., Southwick, A. M. *et al.* (2012a). A genome-wide SNP genotyping array reveals patterns of global and repeated species-pair divergence in sticklebacks. *Curr Biol* 22: 83-90.

Jones, F. C., Grabherr, M. G., Chan, Y. F., Russell, P., Mauceli, E., Johnson, J. *et al.* (2012b). The genomic basis of adaptive evolution in threespine sticklebacks. *Nature* 484: 55-61.

Leblois, R., Gautier, M., Rohfritsch, A., Foucaud, J., Burban, C., Galan, M. *et al*. (2018). Deciphering the demographic history of allochronic differentiation in the pine processionary moth Thaumetopoea pityocampa. *Mol Ecol* 27: 264-278

Magalhaes, I. S., Agostino, D. D., Hohenlohe, P. A. and Maccoll, A. D. C. (2016). The ecology of an adaptive radiation of three-spined stickleback from North Uist, Scotland. *Mol Ecol* 25(17): 4319-4336.

Nei, M. (1973). Analysis of gene diversity in subdivided populations. *Proc Natl Acad Sci USA* 70: 3321-3323.

Nei, M., Li, W-H. (1979). Mathematical model for studying genetic variation in terms of restriction endocleases. *Proc Natl Acad Sci USA* 76: 5269-5273.

Nelson, T. C. and Cresko, W. A. (2018). Ancient genomic variation underlies repeated ecological adaptation in young stickleback populations. *Evol Lett* 2: 9-21.

Roesti, M., Gavrilets, S., Hendry, A. P., Salzburger, W. and Berner, D. (2014). The genomic signature of parallel adaptation from shared genetic variation. *Mol Ecol* 23: 3944-3956.

Roesti, M., Kueng, B., Moser, D. and Berner, D. (2015). The genomics of ecological vicariance in threespine stickleback fish. *Nat Commun* 6 : 8767.

Terekhanova, N.V., Logacheva, M.D., Penin, A.A., Neretina, T.V., Barmintseva A.E., Bazykin, G.A. *et al.* (2014). Fast evolution from precast bricks: genomics of young freshwater populations of threespine stickleback *Gasterosteus aculeatus*. *PLoS Genet* 10: e1004696-e1004696.

Waterston, A. H., A., Campbell, R. and Maitland, P. (1979). Inland waters of the Outer Hebrides. *P Roy Soc Edinb B* 77: 329-351.
